# Leukocyte count and new‐onset diabetes mellitus in people with HIV: A longitudinal study

**DOI:** 10.1111/hiv.70089

**Published:** 2025-08-01

**Authors:** Sophia C. Meyer, Zoe Klein, Isabella C. Schoepf, Christian W. Thorball, Marco Labarile, Neeltje A. Kootstra, Peter Reiss, Lene Ryom, Dominique L. Braun, Maria C. Thurnheer, Catia Marzolini, Christian R. Kahlert, Enos Bernasconi, Matthias Cavassini, Annalisa Marinosci, Jacques Fellay, Huldrych F. Günthard, Roger D. Kouyos, Bruno Ledergerber, Philip E. Tarr, the Swiss HIV Cohort Study, I Abela, K Aebi‐Popp, A Anagnostopoulos, M Battegay, E Bernasconi, DL Braun, HC Bucher, A Calmy, M Cavassini, A Ciuffi, G Dollenmaier, M Egger, L Elzi, J Fehr, J Fellay, H Furrer, CA Fux, HF Günthard, A Hachfeld, D Haerry, B Hasse, HH Hirsch, M Hoffmann, I Hösli, M Huber, D Jackson‐Perry, patient representatives, CR Kahlert, O Keiser, T Klimkait, RD Kouyos, H Kovari, K Kusejko, N Labhardt, K Leuzinger, B Martinez de Tejada, C Marzolini, KJ Metzner, N Müller, J Nemeth, D Nicca, J Notter, P Paioni, G Pantaleo, M Perreau, A Rauch, L Salazar‐Vizcaya, P Schmid, R Speck, M Stöckle, P Tarr, A Trkola, G Wandeler, M Weisser, S. Yerly

**Affiliations:** ^1^ University Center for Internal Medicine and Infectious Diseases Service, Kantonsspital Baselland, Department of Clinical Research University Hospital Basel, University of Basel Bruderholz Switzerland; ^2^ Department of Infectious Diseases Bern University Hospital, University of Bern Bern Switzerland; ^3^ Department of Hepatology, Department for Visceral Surgery and Medicine Bern University Hospital, University of Bern Bern Switzerland; ^4^ Precision Medicine Unit, Lausanne University Hospital and University of Lausanne Lausanne Switzerland; ^5^ Department of Infectious Diseases and Hospital Epidemiology University Hospital Zurich, University of Zurich Zurich Switzerland; ^6^ Institute of Medical Virology University of Zurich Zurich Switzerland; ^7^ Amsterdam University Medical Centers, University of Amsterdam Experimental Immunology Amsterdam The Netherlands; ^8^ Amsterdam University Medical Centers, University of Amsterdam Global Health, and Amsterdam Institute for Global Health and Development Amsterdam The Netherlands; ^9^ Centre of Excellence for Health, Immunity, and Infections, Rigshospitalet, University of Copenhagen Copenhagen Denmark; ^10^ Department of Infectious Diseases Hvidovre University Hospital Copenhagen Denmark; ^11^ Department of Clinical Medicine University of Copenhagen Copenhagen Denmark; ^12^ Division of Infectious Diseases and Hospital Epidemiology University Hospital Basel Basel Switzerland; ^13^ Division of Infectious Diseases Kantonsspital St Gallen St. Gallen Switzerland; ^14^ Division of Infectious Diseases Ente Ospedaliero Cantonale Lugano, University of Geneva and Università della Svizzera italiana Lugano Switzerland; ^15^ Infectious Diseases Service Lausanne University Hospital, University of Lausanne Lausanne Switzerland; ^16^ Division of Infectious Disease Geneva University Hospital Geneva Switzerland; ^17^ School of Life Sciences Ecole Polytechnique Fédérale de Lausanne Lausanne Switzerland

**Keywords:** diabetes mellitus, HIV infection, leukocytes, multivariable analysis, white blood cells

## Abstract

**Objectives:**

The risk of diabetes mellitus (DM) is increased in people with HIV. Chronic inflammation contributes to DM risk. High leukocytes are associated with DM in the general population, but in people with HIV, evidence of a leukocyte–DM association is limited.

**Methods:**

We included participants of the Swiss HIV Cohort Study with new‐onset DM (2000–2023) and matched controls. We obtained uni‐ and multivariable odds ratios (ORs) for DM, based on traditional and HIV‐related DM risk factors, leukocyte count and potential confounders for leukocyte count.

**Results:**

Among 732 DM cases (median age 54 years, 79% male at birth, 86% with suppressed HIV RNA) and 2032 DM‐free controls, the latest leukocyte count prior to DM diagnosis was higher in cases than in controls (median [interquartile range], 6200 [5115–7800] vs. 5900/μL [4800–7180]; *p* < 0.001), but leucocytosis (>11 000/μL) was uncommon (3.8% vs. 2.3%; *p* = 0.032). DM‐OR in the highest vs. lowest leukocyte quintile was 1.91 (95% confidence interval, 1.45–2.52) in univariable analysis and 2.47 (1.71–3.57) in multivariable analysis. For comparison, multivariable DM‐OR for dyslipidaemia, overweight, ≥12‐month stavudine exposure and ≥12‐month integrase inhibitor exposure were 2.23 (1.80–2.75), 2.83 (2.21–3.62), 1.54 (1.16–2.04) and 2.29 (1.13–4.62), respectively. We found no relevant confounders for the leukocyte–DM association. Leukocytes were significantly associated with DM up to 10 years before diagnosis (all *p* < 0.02).

**Conclusions:**

High leukocyte count is an independent DM risk factor in people with HIV in Switzerland and increases the risk of DM to a degree similar to traditional and HIV‐related risk factors, up to 10 years before DM diagnosis.

## BACKGROUND

Multiple studies have recorded an increased risk of type 2 diabetes mellitus (DM) in people with human immunodeficiency virus (HIV) compared to the general population [1, 2, 3, 4]. The pathogenesis of DM in people with HIV may include traditional DM risk factors, most notably general obesity and abdominal obesity [5], and genetic background [6]. In addition, HIV‐related risk factors include the effects of immunosuppression [7, 8], chronic inflammation [9] and the effects of certain antiretroviral therapy (ART) agents [1, 10, 11, 12].

We recently documented an independent association of higher leukocytes with coronary artery disease (CAD) events in people with HIV [13]. Chronic inflammation is also considered a major mechanism in the development of both type 1 and type 2 DM [14, 15, 16] (reviewed in [17]). Elevated leukocytes, mostly within normal range values, are associated with DM risk in the general population, beyond the effects of traditional risk factors such as age, dyslipidaemia and obesity [18, 19, 20, 21]. Information regarding a potential association of leukocytes with DM risk in people with HIV is limited to a report by researchers with the US Women's Interagency HIV Study, who recorded a 1.9% increased DM risk for each 100/μL leukocyte increase [22].

The aim of the present study, therefore, was to examine any association of blood leukocytes with new‐onset DM in participants of the Swiss HIV Cohort Study (SHCS) over a >23‐year observation period, while considering relevant traditional and HIV‐related DM risk factors.

## METHODS

### Study population

Participants were people with HIV enrolled in the SHCS (www.shcs.ch, [23]) having provided written informed consent. The local ethics committees approved the study. Cases had new‐onset DM, and controls were diabetes‐free during the study period, which was 1 January 2000–31 August 2023 (the SHCS began capturing cardiovascular, metabolic, genetic and other data in 1999).

### Diabetes diagnosis

New‐onset DM was defined as suggested by the Expert Committee on the Diagnosis and Classification of Diabetes Mellitus, that is as confirmed plasma glucose ≥7.0 mmol/L (fasting) or ≥11.1 mmol/L (non‐fasting) and typical symptoms or plasma glucose ≥11.1 mmol/L, 2 h after oral intake of 75 g glucose or HbA1c ≥ 6.5% [24]. Participants with prevalent DM or use of DM medication prior to registration in the SHCS were excluded.

### Case–control matching

To account for differences in ART use and other differences during different time periods, we applied a matching procedure, incidence density sampling [25, 26]: We matched controls at the date of DM diagnosis of the corresponding case on similar observation duration and with an observation during similar calendar periods. Matching criteria were sex at birth, date of SHCS registration ±2 years and age at matching date ±2 years. Controls had to be followed up at least until the date of DM diagnosis (matching date) of the corresponding case. Observation time started at SHCS registration and ended for cases at DM diagnosis date and for controls at the first regular SHCS follow‐up visit after the matching date, respectively. We aimed to select one to three non‐diabetic controls for each case.

### Power calculation

Assuming an exposure correlation between pairs in the case–control set of 0.2, we would need 255 cases and 2 controls per case to capture odds ratios (ORs) of ≥1.6 [27].

### Leukocyte count

We considered only leukocytes measured per protocol at 6‐monthly follow‐up SHCS visits up until the matching date. We compared the latest leukocyte count and leukocytes at different intervals before the matching date in cases and controls.

### Diabetes risk factors

As done previously [6, 11, 13, 28, 29], we defined co‐variables a priori, based on their association with DM in the general population, and ascertained them at the last SHCS visit prior to the matching date. Co‐variables included HIV acquisition mode, race/ethnicity (Asian participants stratified by WHO regions [30]), body mass index (BMI [kg/m^2^]; underweight, normal, overweight, obese), abdominal obesity (according to World Health Organization cut‐offs [waist‐hip ratio ≥0.9 for men and ≥0.85 for women] [31, 32]), smoking (never/current/past), hypertension (blood pressure ≥140/90 mmHg or use of antihypertensive medication), dyslipidaemia (total cholesterol >6.2 mmol/L or HDL < 1 mmol/L [men] and <1.2 mmol/L [women] or use of lipid‐lowering drugs [33]), statin treatment [34, 35, 36] and DM family history. HIV‐related co‐variables included viral load (HIV RNA </≥50 copies/mL), CD4 nadir, (CD4 strata <200, 200–349, 350–499, ≥500 cells/μL), history of CDC stage C/AIDS, history of pancreatitis [37, 38], hepatitis C [39] and cytomegalovirus (CMV) seropositivity [40] and type of ART until the matching date. We focused on exposure for a total of one or more years to the ‘third’ agent (i.e., integrase inhibitors [INSTI], boosted protease inhibitors, efavirenz, other non‐nucleoside reverse transcriptase inhibitors [NNRTI] or other) and to thymidine analogues (stavudine, zidovudine) and didanosine and zalcitabine [11, 41, 42].

### Potential confounding variables associated with leukocyte count

We tested each variable in the DM risk model for potential confounding of the leukocyte–DM association and for any interaction with leukocytes (Tables S3 and S4). Because of insufficient available information (e.g., corticosteroid duration/dose/dates, specific diagnoses), we did not analyse corticosteroid use and non‐HIV inflammatory conditions other than infections. Serious non‐opportunistic infections (SNOIs; recorded in the SHCS since 2017, defined as leading to hospitalization or antibiotic use for ≥5 days) and opportunistic infections (OIs) were assessed in cases and controls because of potential effects of infections on leukocyte count.

### Sensitivity analyses

We tested the robustness of the leukocyte–DM association by restricting analysis to (1) white participants, (2) participants with suppressed HIV RNA at matching date, (3) participants without SNOI, (4) participants with available data on family history of DM, (5) participants with available data on physical activity, (6) by considering current (past 6 months) exposure to ART drugs as outlined above [9], (7) by adding any tenofovir (tenofovir disoproxil fumarate [TDF] or tenofovir alafenamide [TAF]) to the model, (8) by restricting analysis to participants without corticosteroid use ≥3 months and (9) by considering statin treatment rather than dyslipidaemia.

### Statistical analyses

We used Fisher's exact test (categorical variables) and Wilcoxon rank‐sum test (continuous variables) to compare characteristics of cases and controls. We used univariable, bivariable and multivariable conditional logistic regression analyses to estimate associations of the different risk factors with DM and their interactions. For better visualization of potentially non‐linear associations with DM, we decided a priori to stratify leukocyte counts into quintiles. We entered variables into the multivariable model if the *p*‐value for their univariable association with DM was <0.2. ART drugs were included if they had a *p*‐value of <0.2 in either of the two codings (≥1‐year total exposure or current use). We used Akaike and Bayesian information criteria and likelihood ratio tests to analyse model fit and interactions. We tested the effect of potential confounders on the leukocyte–DM association on a 1:1 basis (bivariable models including interaction terms). To create trajectories of total leukocytes and HIV RNA over the 15 years prior to the matching date, we used local polynomial smoothing with the Epanechnikov kernel. We used Stata/SE 18.0 (StataCorp, College Station, TX, USA).

## RESULTS

### Participants, diabetes

**FIGURE 1:**
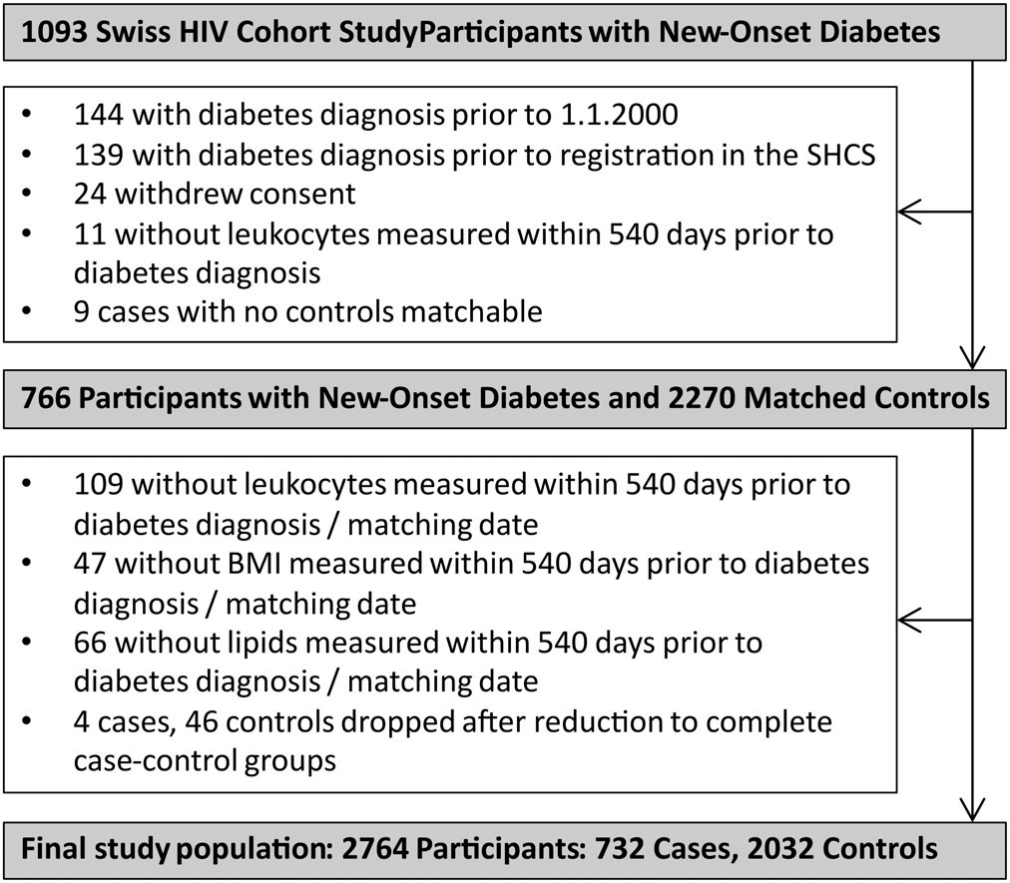
Study flowchart. SHCS, Swiss HIV Cohort Study.

Analyses are based on 2764 participants, that is, 732 cases with new‐onset DM and 2032 matched DM‐free controls. Figure 1 shows participant disposition, Table 1 shows participant characteristics, and detailed information on ART agents is shown in Table S1. Three participants had type 1 DM, and 729 participants had type 2 DM. Median (interquartile range [IQR]) DM diagnosis date was 4 July 2014 (20 May 2008–19 March 2019). Median (IQR) observation duration was 11.6 (6.1–18.5) years and 12.2 (6.9–18.8) years in cases and controls, respectively. Cases were more likely to be Black, of South or West Asian origin, overweight, obese, abdominally obese, dyslipidaemic, treated with a statin, hypertensive, immunosuppressed, HIV‐viraemic and exposed to stavudine, didanosine, zalcitabine or INSTI for ≥12 months. Controls were more likely to be of Southeast Asian origin and treated with TDF or an NNRTI for ≥12 months. There was a trend towards controls being current smokers and cases having a DM family history and exposure to TAF for ≥12 months (Table 1).

**TABLE 1:** Characteristics of cases and controls.

		Participants				Analysis, odds ratio (95% confidence interval) *p*‐value	
		All participants (*n* = 2764)	Cases (*n* = 732)	Controls (*n* = 2032)	*p*‐Value	Univariable	Multivariable
Age (years), median (IQR)^c^		54 (47–61)	54 (47–61)	54 (47–61)	0.687^b^	^c^	^c^
Male sex, *n* (%)^c^ https://www.shcs.ch/health‐care‐providers/		2175 (78.7)	575 (78.6)	1600 (78.7)	0.916^a^	^c^	^c^
Duration of observation (years), median (IQR)		12.1 (6.6–18.7)	11.6 (6.1–18.5)	12.2 (6.9–18.8)	0.079^b^	^c^	^c^
HIV acquisition mode, *n* (%)	MSM	1335 (48.3)	277 (37.8)	1058 (52.1)	<0.001^a^	(Reference)	(Reference)
	IDU	388 (14.0)	100 (13.7)	288 (14.2)		1.50 (1.12–2.03); p = 0.007	1.94 (1.19–3.18); *p* = 0.008
	Heterosexual	927 (33.5)	300 (41.0)	627 (30.9)		2.39 (1.90–3.01); *p* < 0.001	1.46 (1.10–1.94); *p* = 0.009
	Other	114 (4.1)	55 (7.5)	59 (2.9)		4.33 (2.85–6.57); *p* < 0.001	2.83 (1.70–4.73); *p* < 0.001
Ethnicity, *n* (%)	White	2406 (87.1)	578 (79.0)	1828 (90.0)	<0.001^a^	(Reference)	(Reference)
	Black	218 (7.9)	108 (14.8)	110 (5.4)		5.22 (3.62–7.52); *p* < 0.001	3.59 (2.26–5.70); *p* < 0.001
	Hispanic	54 (2.0)	14 (1.9)	40 (2.0)		1.37 (0.73–2.58); *p* = 0.323	1.46 (0.68–3.13); *p* = 0.332
	Asian^d^	86 (3.1)	32 (4.4)	54 (2.7)		2.64 (1.64–4.24); *p* < 0.001	3.71 (2.05–6.72); *p* < 0.001
	South Asia, *n* (% of Asians)	5 (5.8)	4 (4.7)	1 (1.2)			
	Western Asia, *n* (% of Asians)	11	7	4			
	East Asia, *N* (% of Asians)	1	0	1			
	Southeast Asia, *n* (% of Asians)	69 (80.2)	21 (24.4)	48 (55.8)			
BMI (kg/m^2^), median (IQR)		24.4 (21.9–27.5)	27.1 (24.1–31.2)	23.7 (21.5–26.3)		–	–
BMI category, *n* (%)	Underweight	114 (4.1)	22 (3.0)	92 (4.5)	<0.001^a^	1.45 (0.88–2.41); *p* = 0.146	1.26 (0.71–2.22); *p* = 0.431
	Normal	1417 (51.3)	202 (27.6)	1215 (59.8)		(Reference)	(Reference)
	Overweight	866 (31.3)	281 (38.4)	585 (28.8)		3.09 (2.48–3.85); *p* < 0.001	2.83 (2.21–3.62); *p* < 0.001
	Obese	367 (13.3)	227 (31.0)	140 (6.9)		11.81 (8.80–15.86); *p* < 0.001	11.49 (8.14–16.20); *p* < 0.001
Waist‐hip‐ratio, median (IQR) (Available in *n* = 2695 participants)		0.95 (0.90–10)	0.99 (0.94–1.05)	0.94 (0.89–0.99)	<0.001^b^	^e^	^e^
Abdominal obesity, *n* (%) (Available in *n* = 2695 participants)		2309 (85.7)	669 (94.6)	1640 (82.5)	<0.001^a^	4.47 (3.07–6.52); *p* < 0.001	^e^
Smoking status, *n* (%)	Never	910 (32.9)	253 (34.6)	657 (32.3)	0.074^a^	(Reference)	(Reference)
	Current	994 (36.0)	238 (32.5)	756 (37.2)		0.79 (0.64–0.98); *p* = 0.033	0.96 (0.72–1.28); *p* = 0.775
	Past	860 (31.1)	241 (32.9)	619 (30.5)		1.03 (0.83–1.27); *p* = 0.799	1.16 (0.89–1.52); *p* = 0.274
Family history of DM, *n* (%) (Available in *n* = 388 participants)		106 (3.8)	36 (4.9)	70 (3.4)	0.069^a^	1.82 (1.08–3.08); *p* = 0.024	2.91 (1.11–7.67); *p* = 0.030^f^
Hypertension, *n* (%)		850 (30.8)	290 (39.6)	560 (27.6)	<0.001^a^	1.79 (1.49–2.15); *p* < 0.001	1.21 (0.97–1.52); *p* = 0.098
Dyslipidaemia, *n* (%)		1384 (50.1)	492 (67.2)	892 (43.9)	<0.001^a^	2.58 (2.16–3.09); *p* < 0.001	2.23 (1.80–2.75); *p* < 0.001
Statin treatment		216 (7.8)	74 (10.1)	142 (7)	0.008^a^	1.76 (1.25–2.47); *p* < 0.001	1.62 (1.09–2.40); *p* = 0.017^g^
Alcohol use, *n* (%)	None/mild	2062 (87.9)	541 (88.0)	1521 (87.8)	0.943^a^	(Reference)	
	Moderate/heavy	285 (12.1)	74 (12.0)	211 (12.2)		1.00 (0.76–1.33); *p* = 0.981	
Hepatitis C seropositivity, *n* (%)		550 (19.9)	130 (17.8)	420 (20.7)	0.094^a^	0.79 (0.62–1.00); *p* = 0.048	0.77 (0.51–1.16); *p* = 0.214
CMV seropositivity, *n* (%)		2396 (86.7)	630 (86.1)	1766 (86.9)	0.568^a^	0.94 (0.73–1.20); *p* = 0.614	
History of pancreatitis		25 (0.9)	17 (2.3)	8 (0.4)	<0.001^a^	5.97 (2.57–13.89); *p* < 0.001	5.29 (1.90–14.78); *p* = 0.001
Leukocytes/μL, median (IQR)	Latest before DM diagnosis (*n* = 2764)	4049.5 (3550–4400)	4009.5 (3500–4400)	4059.5 (3590–4390)	–	(Reference)	(Reference)
		5105 (4900–5350)	5190 (4900–5300)	5100 (4900–5370)	–	1.10 (0.82–1.46); *p* = 0.533	1.42 (0.99–2.03); *p* = 0.056
		5990 (5780–6200)	6000 (5825–6200)	5960 (5750–6200)	–	1.30 (0.98–1.72); *p* = 0.068	1.65 (1.16–2.35); *p* = 0.006
		6990 (6700–7300)	7000 (6750–7330)	6940 (6700–7300)	–	1.28 (0.96–1.70); *p* = 0.094	1.42 (0.98–2.07); *p* = 0.064
		8900 (8290–10 060)	9100 (8360–10 200)	8800 (8200–9930)	–	1.91 (1.45–2.52); *p* < 0.001	2.47 (1.71–3.57); *p* < 0.001^h^
	Two years before DM diagnosis (*n* = 2645)	–	–	–	–	1.42 (1.07–1.88); *p* = 0.016^h^	–
	Three years before DM diagnosis (*n* = 2528)	–	–	–	–	1.73 (1.29–2.32); *p* < 0.001^h^	–
	Five years before DM diagnosis (*n* = 2281)	–	–	–	–	1.45 (1.08–1.95); *p* = 0.013^g^, ^h^	–
	Eight years before DM diagnosis (*n* = 1896)	–	–	–	–	2.13 (1.50–3.03); *p* < 0.001^h^	–
	Nine years before DM diagnosis (*n* = 1768)	–	–	–	–	1.66 (1.18–2.34); *p* = 0.004 ^h^	–
	Ten years before DM diagnosis (*n* = 1651)	–	–	–	–	1.75 (1.20–2.54); *p* = 0.003^g^, ^h^	–
Leucocytosis (>11 000/mL), *n* (%)		74 (2.7)	28 (3.8)	46 (2.3)	0.032^a^	–	–
CD4 cell count at matching date (cells/μL), median (IQR)		566 (397–785)	577 (366–821)	561 (404–773)	0.924^b^	–	–
CD4 cell count category at matching date (cells/μL), *n* (%)	0–<200	152 (5.5)	56 (7.7)	96 (4.7)	0.004^a^	1.61 (1.12–2.31); *p* = 0.010	2.54 (1.61–4.00); *p* < 0.001
	200–<350	382 (13.8)	113 (15.4)	269 (13.2)		1.16 (0.90–1.50); *p* = 0.251	1.56 (1.13–2.16); *p* = 0.007
	350–<500	572 (20.7)	133 (18.2)	439 (21.6)		0.85 (0.68–1.07); *p* = 0.177	1.12 (0.84–1.50); *p* = 0.429
	≥500	1658 (60.0)	430 (58.7)	1228 (60.4)		(Reference)	(Reference)
CD4 nadir (cells/μL), median (IQR)		167 (70–268)	150 (58–259)	170 (78–271)	0.018^b^	–	–
CD4 nadir <50 cells/μL, *n* (%)		532 (19.3)	164 (22.5)	368 (18.1)	0.012^a^	1.33 (1.08–1.65); *p* = 0.008	–
Previous AIDS, *n* (%)		747 (27.0)	223 (30.5)	524 (25.8)	0.015^a^	1.27 (1.05–1.54); *p* = 0.013	1.11 (0.88–1.41); *p* = 0.376
HIV RNA‐ and ART‐status at matching date, *n* (%)	Interrupted	63 (2.3)	16 (2.2)	47 (2.3)	<0.001^a^	1.00 (0.55–1.80); *p* = 0.992	^i^
	Naïve	84 (3.0)	22 (3.0)	62 (3.1)		0.92 (0.54–1.54); *p* = 0.742	^i^
	On ART, HIV RNA undetectable***	2374 (85.9)	601 (82.1)	1773 (87.3)		(Reference)	^i^
	On ART, HIV RNA detectable***	243 (8.8)	93 (12.7)	150 (7.4)		1.82 (1.35–2.45); *p* < 0.001	^i^
Years on ART, median (IQR)		11.5 (6.3–17.4)	11.4 (6.1–17.4)	11.5 (6.4–17.3)	0.938^b^	–	–
Received third drug ≥1 year in total, *n* (%)	Without ART	142 (5.1)	43 (5.9)	99 (4.9)	0.010^a^	1.25 (0.79–1.97); *p* = 0.347	1.91 (1.06–3.42); *p* = 0.030
	INSTI‐based	119 (4.3)	33 (4.5)	86 (4.2)		1.51 (0.86–2.65); *p* = 0.154	2.29 (1.13–4.62); *p* = 0.021
	Boosted PI‐based	474 (17.2)	106 (14.5)	368 (18.1)		(Reference)	(Reference)
	Efavirenz‐based	441 (16.0)	112 (15.3)	329 (16.2)		1.21 (0.88–1.65); *p* = 0.233	1.61 (1.10–2.34); *p* = 0.014
	NNRTI‐based, other than efavirenz	156 (5.6)	28 (3.8)	128 (6.3)		0.75 (0.46–1.21); *p* = 0.238	0.81 (0.45–1.47); *p* = 0.014
	Other	1432 (51.8)	410 (56.0)	1022 (50.3)		1.52 (1.16–1.97); *p* = 0.002	1.66 (1.21–2.29); *p* = 0.002
Received stavudine ≥1 year in total		896 (32.4)	271 (37.0)	625 (30.8)	0.002^a^	1.45 (1.18–1.79); *p* < 0.001	1.54 (1.16–2.04); *p* = 0.003
Received zidovudine ≥1 year in total		1516 (54.9)	422 (57.7)	1094 (53.8)	0.076 ^a^	1.28 (1.04–1.58); *p* = 0.019	1.41 (1.08–1.83); *p* = 0.011
Received didanosine ≥1 year in total		691 (25)	211 (28.8)	480 (23.6)	0.006 ^a^	1.41 (1.14–1.74); *p* = 0.001	1.26 (0.95–1.68); *p* = 0.105
Received zalcitabine ≥1 year in total		82 (3)	26 (3.6)	56 (2.8)	0.309^a^	1.25 (0.76–2.04); *p* = 0.375	–
Received tenofovir disoproxil fumarate ≥1 year in total		1655 (60)	394 (53.8)	1261 (62.1)	<0.001^a^	0.68 (0.55–0.84); *p* < 0.001	0.65 (0.49–0.87); *p* = 0.003
Received tenofovir alafenamide ≥1 year in total		457 (16.5)	127 (17.4)	330 (16.2)	0.487^a^	1.29 (0.94–1.78); *p* = 0120	1.01 (0.65–1.57); *p* = 0.967
Received lopinavir ≥1 year in total		687 (24.9)	168 (23)	519 (25.5)	0.178^a^	0.89 (0.72–1.10); *p* = 0.291	–
Received indinavir ≥1 year in total		472 (17.1)	134 (18.3)	338 (16.6)	0.303^a^	1.14 (0.89–1.45); *p* = 0.306	–

*Note*: All data shown apply to the matching date and are number (%) of participants, unless otherwise indicated. More details concerning ART drugs of participants are shown in Table S1.

Abbreviations: ART, antiretroviral therapy; CMV, cytomegalovirus; IDU, intravenous drug use; INSTI, integrase inhibitor; IQR, interquartile range; MSM, men who have sex with men; NNRTI, non‐nucleoside reverse transcriptase inhibitors.

^a^ Fisher's exact test.

^b^ Wilcoxon rank‐sum test.

^c^ Age, sex and date of registration were matching criteria; controls had to be followed up at least until the date of DM diagnosis (matching date) of the corresponding case (see Section 2).

^d^ Stratification of Asian participants as per the United Nations M49 (Standard Country or Area Codes for Statistical Use, Series M, No. 49); https://en.wikipedia.org/wiki/UN_M49; no participants originated from ‘Central Asian’ countries.

^e^ Waist‐hip ratio/abdominal obesity not included in final multivariable model because of collinearity with BMI.

^f^ Included in sensitivity analysis (Table S9). Not included in final multivariable model because family history is only available in 388/2764 participants.

^g^ Included in sensitivity analysis (Table S14). Not included in final multivariable model because of collinearity with dyslipidaemia.

^h^ DM‐OR for fifth (highest) versus first (lowest) leukocyte quintile.

^i^ Not included in final multivariable model because of collinearity with current ART (i.e., most patients not on ART have HIV RNA >50 copies/mL).

*** Limit of HIV RNA detection in use at the time of blood draw.

### Latest leukocyte count, observed data

Median (IQR) interval from the latest leukocyte measurement to DM diagnosis (matching date) was 2 (0–43) days in cases vs. 63 (28–109) days in controls. Cases had a higher latest median leukocyte count than controls (median [IQR], 6200 [5115–7800] vs. 5900/μL [4800–7180]; *p* < 0.001; Table 1). Leucocytosis (>11 000/μL) occurred in 3.8% of cases vs. 2.3% of controls (*p* = 0.032). Figure S1 shows how the number of cases increases and the number of controls decreases in higher leukocyte quintiles plus the range of leukocytes in the respective quintiles. Median last leukocyte count was similar in participants treated with a two‐drug antiretroviral regimen (*n* = 116) versus other regimens (*n* = 2648; *p* = 0.42).

### Longitudinal leukocyte values, observed data

**FIGURE 2:**
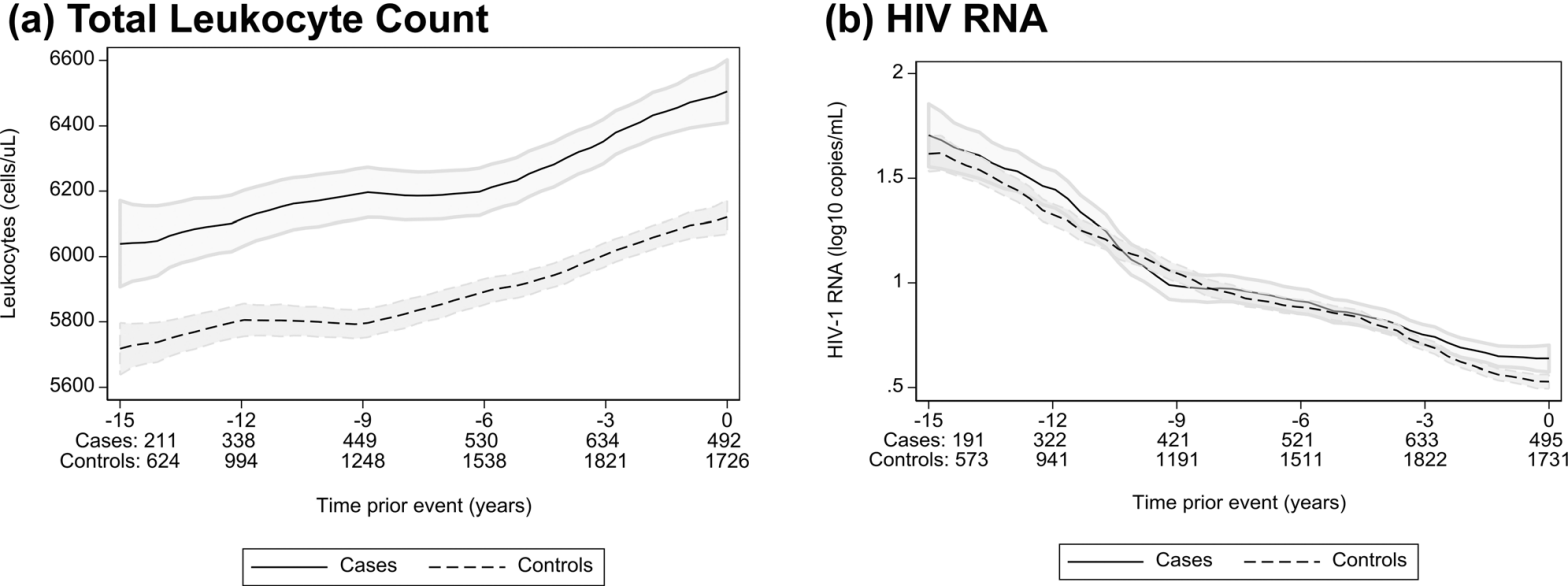
(a, b) Descriptive longitudinal trends for leukocyte count and HIV RNA in cases and controls. Descriptive (observed) trajectories over time for controls versus cases. Panel A shows leukocytes, and panel B shows HIV RNA. Depicted are counts (line) and 95% confidence intervals (shaded areas), which were created with local polynomial smoothing. Only parameters from regular (per protocol) 6‐monthly follow‐up SHCS visits up until the matching date were considered. Data of all participants irrespective of observation duration are shown in the graphs (open cohort design). Graphs for participants with ≥15 years observation time (closed cohort design) are shown in Figure S2A,B.

Figure 2a,b shows longitudinal observed leukocyte and HIV RNA trajectories, respectively. Compared to controls, median (IQR) leukocyte count was higher in cases at year‐2 (*p* = 0.016), year‐3 (*p* < 0.001), year‐5 (*p* = 0.013), year‐8 (*p* < 0.001) and year‐10 (*p* = 0.003) prior to DM diagnosis. HIV RNA values were similar in cases and controls over time.

### Leukocyte count and diabetes, univariable model

**FIGURE 3:**
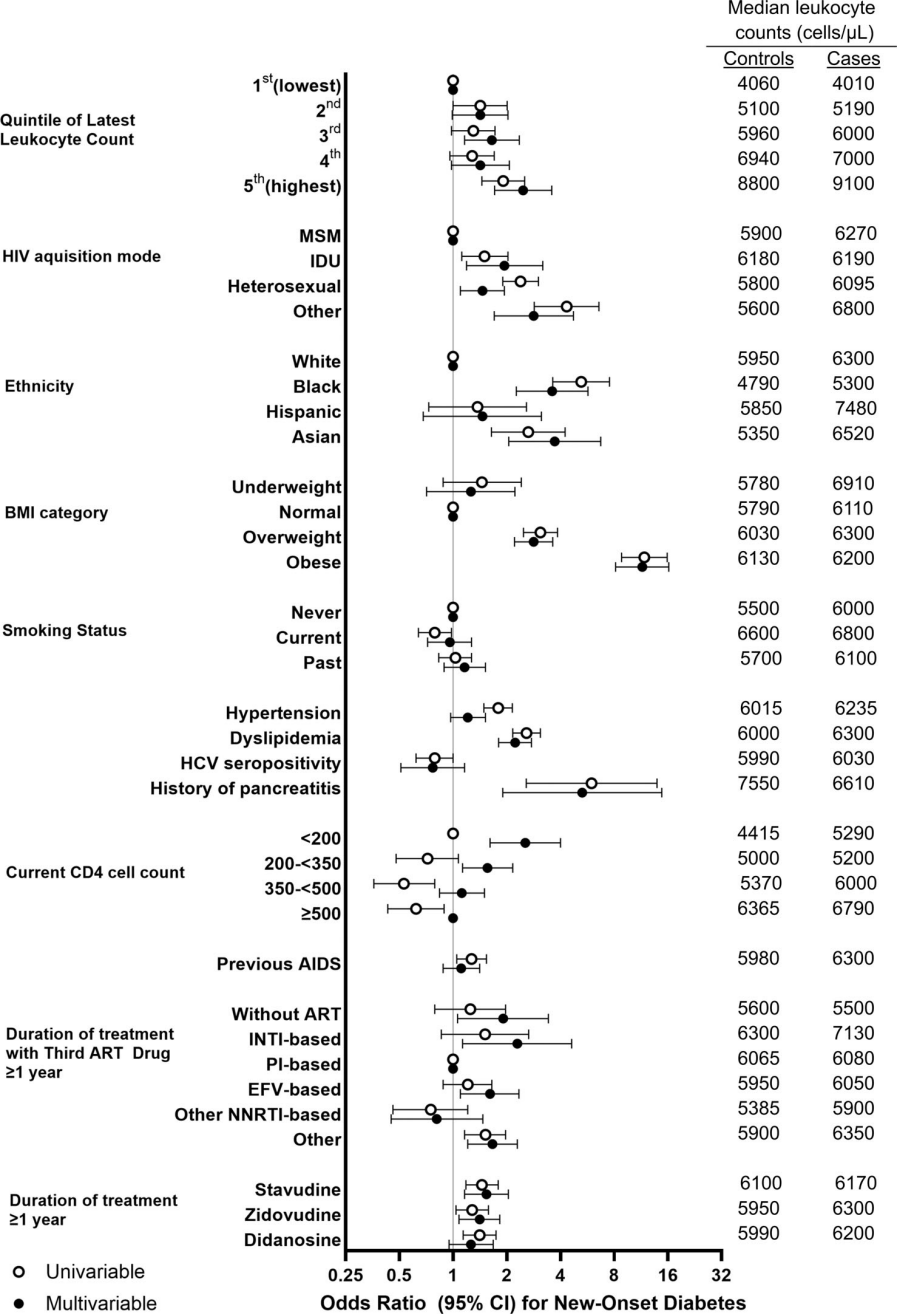
Odds ratios (ORs) for diabetes mellitus (DM; with 95% confidence intervals [CIs]), according to clinical risk factors and latest leukocyte quintiles. Univariable and bivariable conditional logistic regression of associations of latest leukocyte count with new‐onset DM for 732 cases and 2032 controls are shown. DM‐OR was significantly higher in the fifth (highest) than in the first (lowest) leukocyte quintile in univariable and in multivariable analysis, adjusted for all variables shown. Table 1 shows all odds ratios and 95% confidence intervals. Median leukocyte counts (cells/μL) in cases and controls in the different categories are shown on the right‐hand side. ART, antiretroviral therapy; BMI, body mass index; CI, confidence interval; EFV, efavirenz; HCV, hepatitis C virus; IDU, intravenous drug use; INSTI, integrase inhibitor; MSM, men who have sex with men; NNRTI, non‐nucleoside reverse transcriptase inhibitor; PI, protease inhibitor.

At the latest time point prior to the matching date, participants had a 12% increased DM risk per 1000 leukocytes/uL higher (DM‐OR = 1.12, 95% confidence interval [CI], 1.07–1.16). Participants in the second, third, fourth and fifth (highest) quintile had univariable DM‐OR = 1.10 (95% CI, 0.82–1.46), 1.30 (0.98–1.72), 1.28 (0.96–1.70) and 1.91 (1.45–2.52), respectively, compared to participants in the first (lowest) leukocyte quintile. For comparison, univariable DM‐OR for dyslipidaemia, overweight, ≥1‐year stavudine exposure and ≥1‐year INSTI exposure were 2.58 (2.16–3.09), 3.09 (2.48–3.85), 1.45 (1.18–1.79) and 1.48 (1.16–1.89), respectively. Table 1 and Figure 3 show univariable associations of all individual risk factors with DM.

### Longitudinal leukocytes and diabetes, univariable model

Leukocytes (fifth vs. first quintile) were significantly associated with DM when measured at year‐2 (DM‐OR = 1.42; 95% CI, 1.07–1.88; *n* = 2565 participants), year‐3 (1.73; 1.29–2.32; *n* = 2436), year‐5 (1.45; 1.08–1.95; *n* = 2153), year‐8 (2.13; 1.50–3.03; *n* = 1745) and year‐10 (1.75; 1.20–2.54; *n* = 1498) prior to matching date (Table S2).

### Leukocyte count and diabetes, multivariable model

In the final multivariable model, DM risk increased with higher leukocyte count. Compared to the first (lowest) leukocyte quintile, adjusted DM‐OR for participants in the second, third, fourth and fifth quintile was 1.42 (95% CI, 0.99–2.03), 1.65 (1.16–2.35), 1.42 (0.98–2.07) and 2.47 (1.71–3.57), respectively (Table 1, Figure 3). For comparison, multivariable DM‐OR for dyslipidaemia, overweight, ≥1‐year stavudine exposure and ≥1‐year INSTI exposure were 2.23 (1.80–2.75), 2.83 (2.21–3.62), 1.54 (1.16–2.04) and 2.29 (1.13–4.62), respectively.

### Leukocyte count and diabetes, potential confounders

Cases had higher median leukocyte count than controls in all confounder categories (Figure 3). Latest leukocyte count prior to matching date remained associated with DM in each of the 1:1 bivariable analyses (Tables S3 and S4). In bivariable analysis, the effect size of leukocytes on DM‐OR increased when we added ethnicity (fifth vs. first leukocyte quintile, DM‐OR = 2.24 (1.68–2.99); *p* < 0.001), smoking status (fifth vs. first leukocyte quintile, DM‐OR = 2.12 [1.60–2.81]; *p* < 0.001), or CD4 category (fifth vs. first leukocyte quintile, DM‐OR = 2.25 (1.68–3.02); *p* < 0.001). There was minimal confounding for the other individual variables including overweight, obesity and ART agents. Due to lack of significance, all interactions were discarded (Tables S3 and S4). There was a trend (*p* = 0.067) towards an interaction with ethnicity, that is increased DM odds apparent in participants who were Black, South or West Asian (Table 1, Figure 3). However, the leukocyte–DM association persisted and was somewhat attenuated when restricting analysis to the 2072 white participants (adjusted DM‐OR in the fifth vs. first leukocyte quintile, 2.00 [1.31–3.03]; Table S5).

### Leukocyte count and infection episodes

SNOI (recorded in the SHCS since 2017) occurred in 30/279 (10.8%) cases and 55/801 (6.9%) controls (*p* = 0.052). OI occurred in 18 cases and 33 controls (*p* = 0.270; Table S6). Median latest leukocytes were higher in participants with than in those without SNOI (median [IQR], 7140 [5830–9700] vs. 6180 [5180–7500]; *p* < 0.001). Median latest leukocytes were similar in participants with/without OI (*p* = 0.116).

### Sensitivity analyses

Leukocyte count remained significantly associated with DM in all sensitivity analyses, including analysis restricted to the 2105 participants with suppressed viraemia at the latest measurement (Table S7); in the 915 participants without SNOI (Table S8); when DM family history was considered (available in only 388 participants; Table S9); when physical activity was considered (available in 1857 participants; Table S10); when current exposure (past 6 months) to the third ART drug was considered (Table S11); when exposure to TDF or TAF for ≥1 year was considered (Table S12); in analysis restricted to 2260 participants without corticosteroid exposure for ≥3 months (Table S13); and when statin treatment was considered rather than dyslipidaemia (Table S14).

## DISCUSSION

Multiple studies have recorded associations of leukocytes with DM in the general population [18, 19, 20, 21]. In people with HIV, in an analysis of 2592 non‐diabetic US women with HIV followed up for a median duration of 6.6 years, 338 cases of new‐onset DM occurred and a 1.9% increased DM risk per 100 leukocytes/uL higher was recorded [22]. Here, we extend these findings by including all 732 men and women with new‐onset DM enrolled in the well‐established SHCS over a > 23‐year time period. Our six main findings are as follows. First, participants in the fifth (highest) leukocyte quintile had an almost 2.5‐fold increased DM risk compared to participants in the lowest leukocyte quintile in multivariable analysis adjusted for relevant clinical and HIV‐related variables. The effect size of the leukocyte–DM association in people with HIV in Switzerland is similar to effect sizes reported in the general population [18, 19, 20, 21]. Second, the effect of high leukocytes on DM risk was evident up to 10 years prior to DM diagnosis. This is consistent with the direction of the effect being from inflammation to DM in people with HIV and argues against potential reverse causality, as also discussed in meta‐analysis from the general population [19]. Third, results remained significant in multiple sensitivity analyses, and we were unable to identify any variables that attenuated the leukocyte–DM association to any significant degree. Rather, the effect size of leukocytes *increased* in the final multivariable model compared to the univariable model (DM‐OR in the highest leukocyte quintile: 1.91 in univariable analysis, 2.47 in multivariable analysis), reflecting the effects of variables such as immunosuppression, smoking and Black race. Fourth, the DM effect of high leukocytes was larger than the potential adverse effects of certain antiretroviral therapy agents (TAF, INSTI, zidovudine, stavudine), and similar to the effects of well‐known DM risk factors, such as overweight. As expected, the most powerful predictor of DM was obesity. Fifth, high leukocyte counts within normal range values predisposed Swiss people with HIV to DM, and leucocytosis was uncommon, consistent with results in the general population [19]. As expected, infection episodes were associated with increased leukocytes; however, infections were infrequent and did not explain the leukocyte–DM association. Sixth, while adipose tissue is now known as a major contributor to circulating levels of inflammatory biomarkers, the leukocyte–DM association persisted, with only minimal attenuation, after adjusting for obesity, suggesting mostly independent associations of obesity and leukocytes with DM in people with HIV in Switzerland [17, 43].

Our results extend current concepts about how chronic low‐level inflammation in people with HIV contributes to insulin resistance and DM [9]. Importantly, compared to the general population, people with HIV with normal BMI may be at disproportionately increased DM risk [4, 7]. Our findings suggest the potential clinical value of routinely monitoring leukocytes, an inflammatory biomarker that is cheap and widely available in HIV care. Knowledge that high leukocytes, mostly within the normal range, increase DM risk 10 years in advance and by more than twice in 20% of people with HIV (i.e., those in the top leukocyte quintile) may motivate clinicians to place even more emphasis on the optimization of DM risk factors (weight, nutrition, physical activity) and, perhaps, ART selection in such persons. Formal analysis of the clinical value of measuring leukocytes was beyond the scope of our study, however. This will require a prospective clinical trial. Interestingly, chronic inflammation has been linked to accelerated epigenetic ageing in people with HIV [44, 45]. Importantly, in a recent large general population study, elevated leukocytes and elevated neutrophils were associated with accelerated epigenetic ageing [46].

We confirm the known association of Black and South Asian ethnic backgrounds with DM [47, 48, 49]. Even though we recorded a trend towards an interaction of the leukocyte–DM association with ethnicity, the leukocyte effect on DM persisted in the final multivariable model, and in sensitivity analysis restricted to white participants.

Our result of an independent association of leukocytes with DM in people with HIV persisted after adjusting for multiple traditional and HIV‐associated DM risk factors, and in several sensitivity analyses, including multiple different approaches for considering exposure to ART agents with DM association, suggesting the effect is robust. Additional strengths of our study are that all DM diagnoses were validated using internationally standardized criteria [24], data collection was prospective and longitudinal, which allowed us to capture the increase in leukocytes that was apparent already 10 years before DM diagnosis. This, plus the overall rarity of serious infection events prior to DM diagnosis, argues against the leukocyte–DM association in people with HIV being explained by short‐term inflammatory/infectious events. This was confirmed in sensitivity analysis excluding participants with SNOI.

Our study also has limitations. Extrapolation of our results to other people with HIV should be done carefully since our population was mostly male, white and relatively young. Due to insufficient information on chronic inflammatory conditions or corticosteroid use, and because nutrition, leukocyte differential counts or inflammatory markers such as CRP and IL‐6 are not routinely measured in the SHCS, their possible effects on the leukocyte–DM association could not be analysed. HbA1c is not routinely measured in the SHCS, potentially leading to an underestimation of DM cases. Information on physical activity was collected only after 2009, data on non‐ART medication are systematically available only since 2015 and DM family history was available in a minority of participants.

In conclusion, we show that higher leukocytes, mostly within normal range values, are independently associated with new‐onset DM in people with HIV and may help identify persons at increased DM risk up to 10 years before diagnosis. Prospective studies are of considerable interest to determine the clinical value of monitoring leukocytes in predicting DM in diverse groups of PWH.

## AUTHOR CONTRIBUTIONS

Study design: SCM, ZK, BL and PET. Data management, participant selection, case–control matching: BL. Data acquisition: ICS, DLB, MCT, EB, MC, AM, HFG and PET. Data analysis: SCM, ZK, BL and PET. Drafting of the manuscript: SCM, ZK and PET. Critical review and revision of the manuscript: All authors.

## FUNDING INFORMATION

This work was supported by the SHCS (project 836), the Swiss National Science Foundation (grant number 201369) and the SHCS Research Foundation. SHCS data are gathered by the 5 Swiss university hospitals, 2 cantonal hospitals, 15 affiliated hospitals and 36 private physicians (listed in https://www.shcs.ch/health-care-providers/).

## CONFLICT OF INTEREST STATEMENT

PR reports unrestricted scientific grant support from Gilead Sciences, ViiV Healthcare and Merck; consulting fees from Gilead Sciences and ViiV Healthcare; and fees from ViiV Healthcare for chairing and providing an introduction to a workshop; all paid to his institution and unrelated to the submitted work. DLB reports consulting fees from Gilead, MSD and ViiV; personal fees for educational work as well as support for attending meetings from Gilead, MSD and ViiV; and personal fees for participation on a Data Safety Monitoring or Advisory Board from Gilead, MSD, ViiV, AstraZeneca, and Pfizer, outside the submitted work. CM has received personal fees for educational work from Gilead Sciences and ViiV Healthcare outside the submitted work. EB has received grants from the Swiss National Science Foundation and Merck; consulting fees from AstraZeneca; fees for educational work from Pfizer AG Switzerland; support for attending meetings and/or travel from Gilead Sciences, Merck, ViiV Healthcare and Pfizer AG Switzerland; and fees for participation on the Data Safety Monitoring or Advisory Board from Gilead Sciences, ViiV Healthcare, Merck, Pfizer AG Switzerland, AbbVie, AstraZeneca, Moderna and Eli Lilly, all paid to his institution and outside the submitted work. MC reports research grants from Gilead, MSD and ViiV; fees for expert testimony from Gilead, MSD and ViiV; and support for attending meetings from Gilead, all paid to his institution outside the submitted work. HFG has received grants from the Swiss National Science Foundation, Swiss HIV Cohort Study, Yvonne Jacob Foundation, University of Zurich's Clinical Research Priority Program, Zurich Primary HIV Infection, Systems.X, Bill and Melinda Gates Foundation, NIH, Gilead Sciences, ViiV and Roche; personal fees from Merck, Gilead Sciences, ViiV, Janssen, GSK, Johnson & Johnson and Novartis for consultancy or data and safety monitoring board membership; and a travel grant from Gilead. BL received personal fees from Kantonsspital Baselland, Liestal, Switzerland, during the conduct of the study and reports personal fees from Gilead, outside the submitted work. PET's institution reports unrestricted and educational grants from Gilead, ViiV and MSD and advisory fees from Gilead and ViiV, all outside the submitted work. All other authors report no potential conflicts.

## Supporting information


**Data S1.** Supporting information.

## Data Availability

All data for this manuscript were collected in the Swiss HIV Cohort Study.

## APPENDIX A Swiss HIV Cohort Study (SHCS) members

### A.1

Abela I, Aebi‐Popp K, Anagnostopoulos A, Battegay M, Bernasconi E, Braun DL, Bucher HC, Calmy A, Cavassini M, Ciuffi A, Dollenmaier G, Egger M, Elzi L, Fehr J, Fellay J, Furrer H, Fux CA, Günthard HF (President of the SHCS), Hachfeld A, Haerry D (deputy of ‘Positive Council’), Hasse B, Hirsch HH, Hoffmann M, Hösli I, Huber M, Jackson‐Perry D (patient representatives), Kahlert CR (Chairman of the Mother & Child Substudy), Keiser O, Klimkait T, Kouyos RD, Kovari H, Kusejko K (Head of Data Centre), Labhardt N, Leuzinger K, Martinez de Tejada B, Marzolini C, Metzner KJ, Müller N, Nemeth J, Nicca D, Notter J, Paioni P, Pantaleo G, Perreau M, Rauch A (Chairman of the Scientific Board), Salazar‐Vizcaya L, Schmid P, Speck R, Stöckle M (Chairman of the Clinical and Laboratory Committee), Tarr P, Trkola A, Wandeler G, Weisser M, Yerly S.
