# Supplementary material for: Leukocyte count and new‐onset diabetes mellitus in people with HIV: A longitudinal study
Source: HIV Med. 2025 Aug 1;26(10):1585–99. doi: 10.1111/hiv.70089 (PMC12497936; doi:10.1111/hiv.70089)
Supplement: Supplementary file 1 — Data S1. Supporting information. [file HIV-26-1585-s001.docx]

**SUPPLEMENTARY DATA**

Sophia C. Meyer et al

**Leukocyte Count and New-Onset Diabetes Mellitus in People with HIV: A Longitudinal Study**

**Supplementary Results**

**Date of registration, median (IQR)**

All participants: 12 September 1999 (27 August 1995-11 September 2005)

Cases: 19 October 1999 (30 October 1995-31 October 2005)

Controls: 20 August 1999 (25 July 1995-6 September 2005)

P= 0.464

**Matching date, median (IQR)**

All participants: 20 October 2014 (13 August 2008-24 May 2019)

Cases: 4 July 2014 (20 May 2008-19 March 2019)

Controls: 10 November 2014 (17 September 2008-2 July 2019)

P=0.286

**Supplementary Table 1: Antiretroviral Therapy of Cases and Controls**

|  | **All Participants**    (n=2764) | **Cases**  (n=732) | **Controls**  (n=2032) | **P-Values** |
| --- | --- | --- | --- | --- |
| **At least 1 year exposure, n (%)** | | | | |
| Integrase inhibitor | 892 (32.3) | 255 (34.8) | 637 (31.4) | 0.088^a^ |
| Boosted protease inhibitor | 1466 (53.0) | 384 (52.5) | 1082 (53.3) | 0.730^a^ |
| NNRTI other than efavirenz | 709 (25.7) | 178 (24.3) | 531 (26.1) | 0.349^a^ |
| Efavirenz | 1043 (37.7) | 288 (39.3) | 755 (37.2) | 0.307^a^ |
| TDF | 1655 (59.9) | 394 (53.8) | 1261 (62.1) | <0.001^a^ |
| TAF | 457 (16.5) | 127 (17.4) | 330 (16.2) | 0.487^a^ |
| Stavudine | 896 (32.4) | 271 (37.0) | 625 (30.8) | 0.002^a^ |
| Zidovudine | 1516 (54.9) | 422 (57.7) | 1094 (53.8) | 0.076^a^ |
| Didanosine | 691 (25.0) | 211 (28.8) | 480 (23.6) | 0.006^a^ |
| Lopinavir | 687 (24.9) | 168 (23.0) | 519 (25.5) | 0.178^a^ |
| Indinavir | 472 (17.1) | 134 (18.3) | 338 (16.6) | 0.303^a^ |
| **Current (6 months prior to matching date) exposure, n (%)** | | | | |
| Integrase inhibitor | 1001 (36.2) | 289 (39.5) | 712 (35.0) | 0.035^a^ |
| Boosted protease inhibitor | 794 (28.7) | 199 (27.2) | 595 (29.3) | 0.295^a^ |
| NNRTI other than efavirenz | 509 (18.4) | 110 (15.0) | 399 (19.6) | 0.005^a^ |
| Efavirenz | 441 (16.0) | 105 (14.3) | 336 (16.5) | 0.176^a^ |
| TDF | 892 (32.3) | 196 (26.8) | 696 (34.3) | <0.001^a^ |
| TAF | 568 (20.6) | 166 (22.7) | 402 (19.8) | 0.098^a^ |
| **Cumulative years of use, median (IQR)** | | | | |
| Integrase inhibitor | 0 (0-2.40) | 0 (0-2.30) | 0 (0-2.46) | 0.099^b^ |
| Boosted protease inhibitor | 1.39 (0-6.26) | 1.30 (0-6.05) | 1.45 (0-6.34) | 0.343^b^ |
| NNRTI other than efavirenz | 0 (0-1.12) | 0 (0-0.88) | 0 (0-1.22) | 0.688^b^ |
| Efavirenz | 0.02 (0-3.44) | 0.08 (0-3.22) | 0 (0-3.50) | 0.283^b^ |
| TDF | 2.69 (0-6.88) | 1.83 (0-6.31) | 2.93 (0-7.06) | 0.002^b^ |
| TAF | 0 (0-0) | 0 (0-0) | 0 (0-0) | 0.356^b^ |
| Stavudine | 0 (0-2.26) | 0 (0-2.62) | 0 (0-2.15) | 0.005^b^ |
| Zidovudine | 1.62 (0-5.68) | 2.13 (0-5.85) | 1.52 (0-5.59) | 0.047^b^ |

**Note.** All data shown apply to the matching date and are number (%) of participants, unless otherwise indicated.

^a^ Fisher’s exact test

^b^ Wilcoxon rank-sum test

**Abbreviations.** IQR, interquartile range; NNRTI, non-nucleoside reverse transcriptase inhibitor; TAF, tenofovir alafenamide; TDF, tenofovir disoproxil fumarate.

**Supplementary Table 2: Diabetes Odds Ratios and 95% Confidence Intervals According to Leukocyte Count at Different Intervals before Matching Date, Univariable Analysis.**

|  | **Latest**  **Leukocyte Count** | **Year -2**  **Leukocyte Count** | **Year -3**  **Leukocyte Count** | **Year -5**  **Leukocyte Count** |
| --- | --- | --- | --- | --- |
| Definition in time, relative to matching date | 540-0 days before matching date | 541-910 days before matching date | 911-1275 days before matching date | 1645-2005 days before matching date |
| Participants, n | 2764 | 2565 | 2436 | 2153 |
| Odds ratio for DM diagnosis (95% confidence interval),  per 1000 leukocytes higher | 1.12 (1.07-1-16)  p<0.001 | 1.06 (1.01-1.11)  p= 0.010 | 1.10 (1.05-1.15)  p<0.001 | 1.08 (1.03-1.13)  p= 0.002 |
| Odds ratio for DM diagnosis (95% confidence interval),  1st (lowest) leukocyte quintile | (reference) | (reference) | (reference) | (reference) |
| 2nd leukocyte quintile | 1.10 (0.82-1.46)  p= 0.533 | 1.10 (0.82-1.47)  p= 0.529 | 1.14 (0.85-1.54)  p= 0.381 | 0.94 (0.69-1.29)  p= 0.716 |
| 3rd leukocyte quintile | 1.30 (0.98-1.72)  p= 0.068 | 0.91 (0.68-1.22)  p= 0.527 | 1.08 (0.79-1.47)  p= 0.618 | 0.83 (0.61-1.14)  p= 0.252 |
| 4th leukocyte quintile | 1.28 (0.96-1.70)  p= 0.094 | 1.32 (1.00-1.75)  p= 0.052 | 1.49 (1.11-2.00)  p= 0.008 | 0.93 (0.68-1.28)  p= 0.674 |
| 5th (highest) leukocyte quintile | 1.91 (1.45-2.52)  p<0.001 | 1.42 (1.07-1.88)  p= 0.016 | 1.73 (1.29-2.32)  p<0.001 | 1.45 (1.08-1.95)  p= 0.013 |

|  | **Year -8**  **Leukocyte Count** | **Year -9**  **Leukocyte Count** | **Year -10**  **Leukocyte Count** |
| --- | --- | --- | --- |
| Definition in time, relative to matching date | 2740-3100 days before matching date | 3105-3465 days before matching date | 3470-3830 days before matching date |
| Participants, n | 1745 | 1645 | 1498 |
| Odds ratio for DM diagnosis (95% confidence interval),  per 1000 leukocytes higher | 1.11 (1.05-1.17)  p<0.001 | 1.07 (1.01-1.13)  p= 0.013 | 1.11 (1.05-1.18)  p<0.001 |
| Odds ratio for DM diagnosis (95% confidence interval),  1st (lowest) leukocyte quintile | (reference) | (reference) | (reference) |
| 2nd leukocyte quintile | 1.33 (0.92-1.92)  p= 0.135 | 0.88 (0.61-1.26)  p= 0.488 | 0.85 (0.58-1.26)  p= 0.429 |
| 3rd leukocyte quintile | 1.51 (1.04-2.20)  p= 0.032 | 1.18 (0.83-1.70)  p= 0.359 | 1.38 (0.94-2.02)  p= 0.099 |
| 4th leukocyte quintile | 1.95 (1.37-2.77)  p<0.001 | 1.34 (0.94-1.90)  p= 0.101 | 1.67 (1.15-2.43)  p= 0.007 |
| 5th (highest) leukocyte quintile | 2.13 (1.50-3.03)  p<0.001 | 1.66 (1.18-2.34)  p= 0.004 | 1.75 (1.20-2.54)  p= 0.003 |

**Supplementary Table 3: Diabetes Odds Ratios (OR) and 95% Confidence Intervals (CI) According to Leukocyte Quintiles and Clinical Variables, Univariable and Bivariable Analyses Including Potential Interactions (Likelihood-Ratio Test)**

|  | **Univariable Analysis:**  Diabetes Odds Ratios (OR) and 95% Confidence Intervals | **Bivariable**  **Analysis:**  Diabetes Odds Ratios (OR) and 95% Confidence Intervals | **Likelihood-Ratio Test for Interaction** |
| --- | --- | --- | --- |
| **Risk Group:** MSM | (reference) | (reference) | p=0.055 |
| **Risk Group:** IDU | 1.50 (1.12-2.03); p= 0.007 | 1.43 (1.06-1.93); p= 0.020 |  |
| **Risk Group:** heterosexual | 2.39 (1.90-3.01); p<0.001 | 2.34 (1.85-2.95); p<0.001 |  |
| **Risk Group:** other | 4.33 (2.85-6.57); p<0.001 | 4.23 (2.78-6.44); p<0.001 |  |
| 1^st^ (lowest) leukocyte quintile | (reference) | (reference) |  |
| 2nd leukocyte quintile | 1.10 (0.82-1.46); p= 0.533 | 1.14 (0.85-1.53); p= 0.393 |  |
| 3rd leukocyte quintile | 1.30 (0.98-1.72); p= 0.068 | 1.33 (1.00-1.78); p= 0.050 |  |
| 4th leukocyte quintile | 1.28 (0.96-1.70); p= 0.094 | 1.26 (0.94-1.68); p= 0.128 |  |
| 5th (highest) leukocyte quintile | 1.91 (1.45-2.52); p<0.001 | 1.87 (1.41-2.48); p<0.001 |  |
| **Ethnicity:** White | (reference) | (reference) | p=0.067 |
| **Ethnicity:** Black | 5.22 (3.62-7.52); p<0.001 | 5.92 (4.07-8.60); p<0.001 |  |
| **Ethnicity:** Hispanic | 1.37 (0.73-2.58); p= 0.323 | 1.33 (0.70-2.54); p= 0.381 |  |
| **Ethnicity:** Asian | 2.64 (1.64-4.24); p<0.001 | 2.88 (1.78-4.66); p<0.001 |  |
| 1^st^ (lowest) leukocyte quintile | (reference) | (reference) |  |
| 2nd leukocyte quintile | 1.10 (0.82-1.46); p= 0.533 | 1.15 (0.85-1.55); p= 0.370 |  |
| 3rd leukocyte quintile | 1.30 (0.98-1.72); p= 0.068 | 1.45 (1.08-1.94); p= 0.013 |  |
| 4th leukocyte quintile | 1.28 (0.96-1.70); p= 0.094 | 1.43 (1.06-1.93); p= 0.019 |  |
| 5th (highest) leukocyte quintile | 1.91 (1.45-2.52); p<0.001 | 2.24 (1.68-2.99); p<0.001 |  |
| **BMI** Underweight | 1.45 (0.88-2.41); p= 0.146 | 1.45 (0.88-2.41); p= 0.147 | p=0.058 |
| **BMI**: Normal | (reference) | (reference) |  |
| **BMI**: Overweight | 3.09 (2.48-3.85); p<0.001 | 3.02 (2.43-3.76); p<0.001 |  |
| **BMI**: Obese | 11.81 (8.80-15.86); p<0.001 | 11.72 (8.71-15.76); p<0.001 |  |
| 1st (lowest) leukocyte quintile | (reference) | (reference) |  |
| 2nd leukocyte quintile | 1.10 (0.82-1.46); p= 0.533 | 1.15 (0.84-1.59); p= 0.385 |  |
| 3rd leukocyte quintile | 1.30 (0.98-1.72); p= 0.068 | 1.33 (0.98-1.82); p= 0.071 |  |
| 4th leukocyte quintile | 1.28 (0.96-1.70); p= 0.094 | 1.16 (0.84-1.60); p= 0.367 |  |
| 5th (highest) leukocyte quintile | 1.91 (1.45-2.52); p<0.001 | 1.81 (1.33-2.46); p<0.001 |  |
| **Abdominal Obesity**  (Available in n= 2629 participants) | 4.47 (3.07-6.52); p<0.001 | 4.50 (3.08-6.58); p<0.001 | p<0.001 |
| 1st (lowest) leukocyte quintile | (reference) | (reference) |  |
| 2nd leukocyte quintile | 1.06 (0.79-1.41); p= 0.713 | 1.08 (0.80-1.46); p= 0.594 |  |
| 3rd leukocyte quintile | 1.26 (0.95-1.68); p= 0.108 | 1.25 (0.93-1.67); p= 0.141 |  |
| 4th leukocyte quintile | 1.25 (0.93-1.67); p= 0.140 | 1.24 (0.91-1.67); p= 0.168 |  |
| 5th (highest) leukocyte quintile | 1.84 (1.39-2.43); p<0.001 | 1.88 (1.41-2.50); p<0.001 |  |
| **Smoking:** Never | (reference) | (reference) | p=0.571 |
| **Smoking:** Current smoking | 0.79 (0.64-0.98); p= 0.033 | 0.70 (0.56-0.88); p= 0.002 |  |
| **Smoking:** Past smoking | 1.03 (0.83-1.27); p= 0.799 | 1.03 (0.83-1.28); p= 0.776 |  |
| 1^st^ (lowest) leukocyte quintile | (reference) | (reference) |  |
| 2^nd^ leukocyte quintile | 1.10 (0.82-1.46); p= 0.533 | 1.10 (0.83-1.47); p= 0.506 |  |
| 3rd leukocyte quintile | 1.30 (0.98-1.72); p= 0.068 | 1.32 (1.00-1.75); p= 0.052 |  |
| 4th leukocyte quintile | 1.28 (0.96-1.70); p= 0.094 | 1.37 (1.03-1.83); p= 0.033 |  |
| 5th (highest) leukocyte quintile | 1.91 (1.45-2.52); p<0.001 | 2.12 (1.60-2.81); p<0.001 |  |
| **Family History of DM**  (Available in n=388 participants) | 1.82 (1.08-3.08); p= 0.024 | 2.03 (1.18-3.48); p= 0.010 | p=0.170 |
| 1st (lowest) leukocyte quintile | (reference) | (reference) |  |
| 2nd leukocyte quintile | 0.91 (0.42-1.98); p= 0.816 | 0.96 (0.44-2.12); p= 0.929 |  |
| 3rd leukocyte quintile | 1.77 (0.82-3.80); p= 0.145 | 1.99 (0.91-4.37); p= 0.085 |  |
| 4th leukocyte quintile | 1.17 (0.56-2.44); p= 0.681 | 1.22 (0.57-2.59); p= 0.611 |  |
| 5th (highest) leukocyte quintile | 2.66 (1.31-5.41); p=0.007 | 2.94 (1.41-6.12) p= 0.004 |  |
| **Hypertension** | 1.79 (1.49-2.15); p<0.001 | 1.77 (1.47-2.13); p<0.001 | p=0.635 |
| 1st (lowest) leukocyte quintile | (reference) | (reference) |  |
| 2nd leukocyte quintile | 1.10 (0.82-1.46); p= 0.533 | 1.07 (0.80-1.43); p= 0.647 |  |
| 3rd leukocyte quintile | 1.30 (0.98-1.72); p= 0.068 | 1.24 (0.93-1.65); p= 0.135 |  |
| 4th leukocyte quintile | 1.28 (0.96-1.70); p= 0.094 | 1.21 (0.90-1.61); p= 0.206 |  |
| 5th (highest) leukocyte quintile | 1.91 (1.45-2.52); p<0.001 | 1.85 (1.41-2.44); p<0.001 |  |
| **Dyslipidemia** | 2.58 (2.16-3.09); p<0.001 | 2.57 (2.14-3.08); p<0.001 | p=0.720 |
| 1st (lowest) leukocyte quintile | (reference) | (reference) |  |
| 2nd leukocyte quintile | 1.10 (0.82-1.46); p= 0.533 | 1.14 (0.85-1.54); p= 0.375 |  |
| 3rd leukocyte quintile | 1.30 (0.98-1.72); p= 0.068 | 1.28 (0.96-1.70); p= 0.098 |  |
| 4th leukocyte quintile | 1.28 (0.96-1.70); p= 0.094 | 1.22 (0.91-1.64); p= 0.188 |  |
| 5th (highest) leukocyte quintile | 1.91 (1.45-2.52); p<0.001 | 1.89 (1.43-2.51); p<0.001 |  |
| **Last alcohol intake:**  (Available in n=2339 participants)  none/mild | (reference) | (reference) | p=0.348 |
| **Last alcohol intake:** moderate/heavy | 1.00 (0.76-1.33); p= 0.981 | 0.98 (0.74-1.31); p= 0.899 |  |
| 1st (lowest) leukocyte quintile | (reference) | (reference) |  |
| 2nd leukocyte quintile | 1.02 (0.74-1.41); p= 0.903 | 1.02 (0.74-1.41); p= 0.907 |  |
| 3rd leukocyte quintile | 1.28 (0.95-1.74); p= 0.110 | 1.28 (0.94-1.74); p= 0.111 |  |
| 4th leukocyte quintile | 1.22 (0.89-1.67); p= 0.218 | 1.22 (0.89-1.67); p= 0.218 |  |
| 5th (highest) leukocyte quintile | 1.91 (1.41-2.57); p<0.001 | 1.91 (1.41-2.57); p<0.001 |  |
| **Hepatitis C seropositivity** | 0.79 (0.62-1.00); p= 0.048 | 0.78 (0.61-0.99); p= 0.039 | p=0.561 |
| 1st (lowest) leukocyte quintile | (reference) | (reference) |  |
| 2nd leukocyte quintile | 1.10 (0.82-1.46); p= 0.533 | 1.08 (0.81-1.45); p= 0.587 |  |
| 3rd leukocyte quintile | 1.30 (0.98-1.72); p= 0.068 | 1.29 (0.98-1.71); p= 0.073 |  |
| 4th leukocyte quintile | 1.28 (0.96-1.70); p= 0.094 | 1.27 (0.95-1.70); p= 0.102 |  |
| 5th (highest) leukocyte quintile | 1.91 (1.45-2.52); p<0.001 | 1.91 (1.45-2.52); p<0.001 |  |
| **CMV seropositivity** | 0.94 (0.73-1.20); p= 0.614 | 0.96 (0.74-1.23); p= 0.731 | p=0.314 |
| 1st (lowest) leukocyte quintile | (reference) | (reference) |  |
| 2nd leukocyte quintile | 1.10 (0.82-1.46); p= 0.533 | 1.10 (0.82-1.46); p= 0.537 |  |
| 3rd leukocyte quintile | 1.30 (0.98-1.72); p= 0.068 | 1.30 (0.98-1.72); p= 0.069 |  |
| 4th leukocyte quintile | 1.28 (0.96-1.70); p= 0.094 | 1.28 (0.96-1.70); p= 0.095 |  |
| 5th (highest) leukocyte quintile | 1.91 (1.45-2.52); p<0.001 | 1.91 (1.45-2.51); p<0.001 |  |
| **History of pancreatitis** | 5.97 (2.57-13.89); p<0.001 | 5.54 (2.36-13.01); p<0.001 | p= 0.103 |
| 1st (lowest) leukocyte quintile | (reference) | (reference) |  |
| 2nd leukocyte quintile | 1.10 (0.82-1.46); p= 0.533 | 1.11 (0.83-1.48); p= 0.478 |  |
| 3rd leukocyte quintile | 1.30 (0.98-1.72); p= 0.068 | 1.29 (0.97-1.71); p= 0.079 |  |
| 4th leukocyte quintile | 1.28 (0.96-1.70); p= 0.094 | 1.27 (0.95-1.70); p= 0.100 |  |
| 5th (highest) leukocyte quintile | 1.91 (1.45-2.52); p<0.001 | 1.89 (1.43-2.49); p<0.001 |  |
| **CD4 cell count category at matching date**: <200 | 1.61 (1.12-2.31); p=0.010 | 2.05 (1.40-3.01); p<0.001 | p= 0.945 |
| **CD4 cell count category at matching date**: 200-<350 cells/μL | 1.16 (0.90-1.50); p= 0.251 | 1.43 (1.10-1.87); p= 0.009 |  |
| **CD4 cell count category at matching date**: 350-<500 cells/μL | 0.85 (0.68-1.07); p= 0.177 | 0.98 (0.78-1.25); p=0.900 |  |
| **CD4 cell count category at matching date**: ≥500 cells/μL | (reference) | (reference) |  |
| 1st (lowest) leukocyte quintile | (reference) | (reference) |  |
| 2nd leukocyte quintile | 1.10 (0.82-1.46); p= 0.533 | 1.22 (0.91-1.64); p= 0.189 |  |
| 3rd leukocyte quintile | 1.30 (0.98-1.72); p= 0.068 | 1.47 (1.10-1.97); p= 0.010 |  |
| 4th leukocyte quintile | 1.28 (0.96-1.70); p= 0.094 | 1.48 (1.10-2.01); p= 0.011 |  |
| 5th (highest) leukocyte quintile | 1.91 (1.45-2.52); p<0.001 | 2.25 (1.68-3.02); p<0.001 |  |
| **AIDS** | 1.27 (1.05-1.54); p= 0.013 | 1.27 (1.05-1-53); p=0.016 | p= 0.397 |
| 1st (lowest) leukocyte quintile | (reference) | (reference) |  |
| 2nd leukocyte quintile | 1.10 (0.82-1.46); p= 0.533 | 1.12 (0.84-1.50); p=0.439 |  |
| 3rd leukocyte quintile | 1.30 (0.98-1.72); p= 0.068 | 1.33 (1.00-1.76); p= 0.048 |  |
| 4th leukocyte quintile | 1.28 (0.96-1.70); p= 0.094 | 1.29 (0.97-1.72); p= 0.084 |  |
| 5th (highest) leukocyte quintile | 1.91 (1.45-2.52); p<0.001 | 1.92 (1.46-2.53); p<0.001 |  |
| **Received Third ART Drug ≥1 year in total**: without ART | 1.25 (0.79-1.97); p= 0.347 | 1.28 (0.81-2.03); p= 0.293 | p= 0.246 |
| **Received Third ART Drug ≥1 year in total**: Integrase inhibitor | 1.51 (0.86-2.65); p= 0.154 | 1.51 (0.86-2.66); p= 0.155 |  |
| **Received Third ART Drug ≥1 year in total**: Boosted protease inhibitor | (reference) | (reference) |  |
| **Received Third ART Drug ≥1 year in total**: Efavirenz | 1.21 (0.88-1.65); p= 0.233 | 1.21 (0.88-1.66); p= 0.234 |  |
| **Received Third ART Drug ≥1 year in total**: NNRTI other than efavirenz | 0.75 (0.46-1.21); p= 0.238 | 0.79 (0.49-1.28); p= 0.337 |  |
| **Received Third ART Drug ≥1 year in total**: Other | 1.52 (1.16-1.97); p= 0.002 | 1.55 (1.19-2.02); p= 0.001 |  |
| 1st (lowest) leukocyte quintile | (reference) | (reference) |  |
| 2nd leukocyte quintile | 1.10 (0.82-1.46); p= 0.533 | 1.11 (0.83-1.48); p= 0.482 |  |
| 3rd leukocyte quintile | 1.30 (0.98-1.72); p= 0.068 | 1.30 (0.98-1.73); p= 0.066 |  |
| 4th leukocyte quintile | 1.28 (0.96-1.70); p= 0.094 | 1.28 (0.96-1.72); p= 0.092 |  |
| 5th (highest) leukocyte quintile | 1.91 (1.45-2.52); p<0.001 | 1.93 (1.46-2.54); p<0.001 |  |
| **Received Stavudine at least 1 year in total** | 1.45 (1.18-1.79); p<0.001 | 1.41 (1.14-1.75); p= 0.001 | p=0.389 |
| 1st (lowest) leukocyte quintile | (reference) | (reference) |  |
| 2nd leukocyte quintile | 1.10 (0.82-1.46); p= 0.533 | 1.10 (0.82-1.46); p= 0.536 |  |
| 3rd leukocyte quintile | 1.30 (0.98-1.72); p= 0.068 | 1.29 (0.98-1.72); p= 0.072 |  |
| 4th leukocyte quintile | 1.28 (0.96-1.70); p= 0.094 | 1.29 (0.97-1.72); p= 0.083 |  |
| 5th (highest) leukocyte quintile | 1.91 (1.45-2.52); p<0.001 | 1.88 (1.42-2.47); p<0.001 |  |
| **Received Zidovudine at least 1 year in total** | 1.28 (1.04-1.58); p= 0.019 | 1.27 (1.03-1.56); p= 0.023 | p=0.471 |
| 1st (lowest) leukocyte quintile | (reference) | (reference) |  |
| 2nd leukocyte quintile | 1.10 (0.82-1.46); p= 0.533 | 1.09 (0.82-1.45); p= 0.568 |  |
| 3rd leukocyte quintile | 1.30 (0.98-1.72); p= 0.068 | 1.29 (0.98-1.72); p= 0.073 |  |
| 4th leukocyte quintile | 1.28 (0.96-1.70); p= 0.094 | 1.27 (0.95-1.69); p= 0.107 |  |
| 5th (highest) leukocyte quintile | 1.91 (1.45-2.52); p<0.001 | 1.90 (1.44-2.50); p<0.001 |  |
| **Received Didanosine at least 1 year in total** | 1.41 (1.14-1.74); p= 0.001 | 1.41 (1.14-1.74); p= 0.002 | p= 0.960 |
| 1st (lowest) leukocyte quintile | (reference) | (reference) |  |
| 2nd leukocyte quintile | 1.10 (0.82-1.46); p= 0.533 | 1.10 (0.83-1.47); p= 0.510 |  |
| 3rd leukocyte quintile | 1.30 (0.98-1.72); p= 0.068 | 1.30 (0.98-1.72); p= 0.068 |  |
| 4th leukocyte quintile | 1.28 (0.96-1.70); p= 0.094 | 1.28 (0.96-1.70); p= 0.097 |  |
| 5th (highest) leukocyte quintile | 1.91 (1.45-2.52); p<0.001 | 1.92 (1.46-2.52); p<0.001 |  |
| **Received Zalcitabine at least 1 year in total** | 1.25 (0.76-2.04); p= 0.375 | 1.22 (0.74-2.01); p= 0.431 | p= 0.867 |
| 1st (lowest) leukocyte quintile | (reference) | (reference) |  |
| 2nd leukocyte quintile | 1.10 (0.82-1.46); p= 0.533 | 1.09 (0.82-1.46); p= 0.555 |  |
| 3rd leukocyte quintile | 1.30 (0.98-1.72); p= 0.068 | 1.30 (0.98-1.72); p= 0.072 |  |
| 4th leukocyte quintile | 1.28 (0.96-1.70); p= 0.094 | 1.27 (0.95-1.70); p= 0.100 |  |
| 5th (highest) leukocyte quintile | 1.91 (1.45-2.52); p<0.001 | 1.91 (1.45-2.51); p= 0.431 |  |

**Abbreviations.** ART, antiretroviral therapy, BMI, body mass index; confidence interval; CMV, cytomegalovirus; DM diabetes mellitus; HCV, Hepatitis C Virus; IDU, intravenous drug use; NNRTI, non-nucleoside reverse transcriptase inhibitor; OR, Odds Ratio

**Supplementary Table 4: Bivariable Analyses Showing Diabetes Odds Ratio (95% Confidence Interval) for Fifth (Highest) vs. First (Lowest) Leukocyte Quintile, with 1:1 Addition of Individual Variables that May Influence Leukocyte Count**

| **Variable** | **Diabetes odds ratio (95% confidence interval)**  for 5^th^ (highest) vs. 1^st^ (lowest) leukocyte quintile | **Likelihood ratio test for Interaction** |
| --- | --- | --- |
| **Univariable Analysis** | | |
| Leukocytes, 5^th^ (highest) vs.  1^st^ (lowest) quintile | 1.91 (1.45-2.52) | -- |
| **Individual Bivariable Analyses (Leukocyte Quintiles plus Individual Variables added 1:1)** | | |
| + Ethnicity | 2.24 (1.68-2.99) | 0.067 |
| + Smoking status | 2.12 (1.60-2.81) | 0.571 |
| + CD4 category | 2.25 (1.68-3.02) | 0.945 |

**Supplementary Table 5: Sensitivity Analysis: Diabetes Odds Ratio (95% Confidence Interval) Restricted to Participants with White Ethnicity (2072/2764 study participants; 571 cases, 1828 controls)**

|  | **Multivariable analysis:**  **Diabetes Odds Ratios (OR) and 95% Confidence Intervals** |
| --- | --- |
| 1^st^ (lowest) leukocyte quintile* | (reference) |
| 2nd leukocyte quintile* | 1.32 (0.87-2.00); p= 0.195 |
| 3rd leukocyte quintile* | 1.55 (1.04-2.30); p= 0.033 |
| 4th leukocyte quintile* | 1.23 (0.81-1.87); p= 0.339 |
| 5th (highest) leukocyte quintile* | 2.00 (1.31-3.03); p= 0.001 |
| **HIV acquisition mode:** MSM | (reference) |
| **HIV acquisition mode:** IDU | 1.89 (1.11-3.21); p= 0.019 |
| **HIV acquisition mode:** Heterosexual | 1.32 (0.98-1.80); p= 0.071 |
| **HIV acquisition mode:** Other | 2.67 (1.47-4.84); p= 0.001 |
| **Ethnicity** | (omitted) |
| **BMI:** Underweight | 1.27 (0.69-2.36); p= 0.442 |
| **BMI:** Normal | (reference) |
| **BMI:** Overweight | 2.85 (2.17-3.75); p<0.001 |
| **BMI:** Obese | 10.27 (6.99-15.09); p<0.001 |
| **Smoking status**: Never | (reference) |
| **Smoking status**: Current | 1.07 (0.77-1.48); p= 0.689 |
| **Smoking status**: Past | 1.23 (0.91-1.65); p= 0.177 |
| **Hypertension** | 1.25 (0.97-1.59); p= 0.081 |
| **Dyslipidemia** | 2.16 (1.70-2.73); p<0.001 |
| **Hepatitis C seropositivity** | 0.76 (0.49-1.19); p= 0.231 |
| **History of pancreatitis** | 4.06 (1.37-12.00); p= 0.011 |
| **CD4 cell count category**: 0-<200 cells/μL | 2.37 (1.44-3.92); p= 0.001 |
| **CD4 cell count category**: 200-<350 cells/μL | 1.53 (1.07-2.18); p= 0.020 |
| **CD4 cell count category**: 350-<500 cells/μL | 1.04 (0.75-1.44); p= 0.798 |
| **CD4 cell count category**: ≥500 cells/μL | (reference) |
| **Previous AIDS** | 1.13 (0.87-1.47); p= 0.347 |
| **Received third drug ≥1 year in total**: Without ART | 1.44 (0.74-2.81); p= 0.286 |
| **Received third drug ≥1 year in total**: Integrase inhibitor | 1.98 (0.85-4.57); p= 0.112 |
| **Received third drug ≥1 year in total**: Boosted protease inhibitor | (reference) |
| **Received third drug ≥1 year in total**: Efavirenz | 1.49 (0.98-2.27); p= 0.065 |
| **Received third drug ≥1 year in total**: NNRTI other than efavirenz | 0.85 (0.44-1.64); p= 0.627 |
| **Received third drug ≥1 year in total**: Other | 1.59 (1.12-2.25); p= 0.009 |
| **Received stavudine ≥1 year in total** | 1.55 (1.14-2.11); p= 0.005 |
| **Received zidovudine ≥1 year in total** | 1.40 (1.05-1.88); p= 0.022 |
| **Received didanosine ≥1 year in total** | 1.18 (0.87-1.60); p= 0.285 |

**Note.** All data shown apply to the matching date and are number (%) of participants, unless otherwise indicated.

**Abbreviations.** ART, antiretroviral therapy; IDU, intravenous drug use; MSM, men who have sex with men; NNRTI, non-nucleoside reverse transcriptase inhibitor

* latest leukocyte count before matching date

**Supplementary Table 6: Opportunistic Infections and Serious Non-Opportunistic Infections in Cases and Controls.**

| **Category** | **Cases, n (%)** | **Controls, n (%)** | **p-value** | **Latest Leukocyte Count, median (IQR), cells/uL**  **All participants** | **p-value** |
| --- | --- | --- | --- | --- | --- |
| Opportunistic infections * | | | | | |
| Without (n=2159) | 602 (97.1) | 1557 (97.9) | 0.270^a^ | 6000 (5000-7400) | 0.116^b^ |
| With (n=51) | 18 (2.9) | 33 (2.1) |  | 5700 (4190-7560) |  |
| Total (n=2210) | 620 (100) | 1590 (100) |  | 6000 (4960-7400) |  |
| Serious non-opportunistic infections ** | | | | | |
| Without (n=743) | 199 (88.4) | 544 (92.7) | 0.067^a^ | 6180 (5180-7500) | <0.001^b^ |
| With (n=69) | 26 (11.6) | 43 (7.3) |  | 7140 (5830-9700) |  |
| Total (n=812) | 225 (100) | 587 (100) |  | 6200 (5200-7590) |  |

**Abbreviations:** IQR, interquartile range.

* defined as CDC Stage B or C events

** captured in SHCS database since September 2017

^a^ Fisher’s exact Test

^b^ Wilcoxon rank-sum Test

**Supplementary Table 7: Sensitivity Analysis: Diabetes Odds Ratio (95% Confidence Interval) Restricted to Participants with Suppressed HIV RNA (2105/2764 study participants; 589 cases, 1516 controls)**

|  | **Multivariable analysis:**  **Diabetes Odds Ratios (OR) and 95% Confidence Intervals** |
| --- | --- |
| 1^st^ (lowest) leukocyte quintile* | (reference) |
| 2nd leukocyte quintile* | 1.33 (0.87-2.05); p= 0.188 |
| 3rd leukocyte quintile* | 1.58 (1.04-2.40); p= 0.034 |
| 4th leukocyte quintile* | 1.32 (0.85-2.05); p= 0.213 |
| 5th (highest) leukocyte quintile* | 2.47 (1.59-3.82); p<0.001 |
| **HIV acquisition mode:** MSM | (reference) |
| **HIV acquisition mode:** IDU | 1.90 (1.09-3.32); p= 0.023 |
| **HIV acquisition mode:** Heterosexual | 1.31 (0.94-1.82); p= 0.113 |
| **HIV acquisition mode:** Other | 2.38 (1.34-4.22); p= 0.003 |
| **Ethnicity:** White | 2.69 (1.56-4.64); p<0.001 |
| **Ethnicity:** Black | 2.69 (1.56-4.64); p<0.001 |
| **Ethnicity:** Hispanic | 1.70 (0.69-4.20); p= 0.253 |
| **Ethnicity:** Asian | 3.57 (1.81-7.06); p<0.001 |
| **BMI:** Underweight | 1.14 (0.59-2.21); p= 0.694 |
| **BMI:** Normal | (reference) |
| **BMI:** Overweight | 3.35 (2.49-4.50); p<0.001 |
| **BMI:** Obese | 15.00 (9.88-22.77); p<0.001 |
| **Smoking status**: Never | (reference) |
| **Smoking status**: Current | 1.07 (0.76-1.49); p= 0.710 |
| **Smoking status**: Past | 1.18 (0.87-1.61); p= 0.288 |
| **Hypertension** | 1.30 (0.87-1.61); p= 0.288 |
| **Dyslipidemia** | 2.29 (1.79-2.93); p<0.001 |
| **Hepatitis C seropositivity** | 0.89 (0.56-1.41); p= 0.618 |
| **History of pancreatitis** | 5.62 (1.59-19.84); p= 0.007 |
| **CD4 cell count category**: 0-<200 cells/μL | 2.54 (1.38-4.69); p= 0.003 |
| **CD4 cell count category**: 200-<350 cells/μL | 1.84 (1.23-2.74); p= 0.003 |
| **CD4 cell count category**: 350-<500 cells/μL | 1.10 (0.79-1.54); p= 0.577 |
| **CD4 cell count category**: ≥500 cells/μL | (reference) |
| **Previous AIDS** | 1.12 (0.85-1.47); p= 0.428 |
| **Received third drug ≥1 year in total**: Without ART | 1.88 (0.53-6.65); p= 0.326 |
| **Received third drug ≥1 year in total**: Integrase inhibitor | 2.19 (1.03-4.64); p= 0.041 |
| **Received third drug ≥1 year in total**: Boosted protease inhibitor | (reference) |
| **Received third drug ≥1 year in total**: Efavirenz | 1.60 (1.05-2.43); p= 0.028 |
| **Received third drug ≥1 year in total**: NNRTI other than efavirenz | 0.67 (0.34-1.32); p= 0.249 |
| **Received third drug ≥1 year in total**: Other | 1.46 (1.02-2.10); p= 0.039 |
| **Received stavudine ≥1 year in total** | 1.74 (1.25-2.42); p= 0.001 |
| **Received zidovudine ≥1 year in total** | 1.53 (1.13-2.08); p= 0.006 |
| **Received didanosine ≥1 year in total** | 1.32 (0.95-1.84); p= 0.100 |

**Note.** All data shown apply to the matching date and are number (%) of participants, unless otherwise indicated.

**Abbreviations.** ART, antiretroviral therapy; IDU, intravenous drug use; MSM, men who have sex with men; NNRTI, non-nucleoside reverse transcriptase inhibitor

* latest leukocyte count before matching date

**Supplementary Table 8: Sensitivity Analysis: Diabetes Odds Ratio (95% Confidence Interval) Restricted to Participants without Serious Non-Opportunistic Infection (since 01/2017, 915/2764 study participants; 247 cases, 668 controls)**

|  | **Multivariable analysis:**  **Diabetes Odds Ratios (OR) and 95% Confidence Intervals** |
| --- | --- |
| 1^st^ (lowest) leukocyte quintile* | (reference) |
| 2nd leukocyte quintile* | 2.10 (0.97-4.52); p= 0.059 |
| 3rd leukocyte quintile* | 2.73 (1.27-5.85); p= 0.010 |
| 4th leukocyte quintile* | 1.99 (0.94-4.21); p= 0.072 |
| 5th (highest) leukocyte quintile* | 4.78 (2.21-10.35); p<0.001 |
| **HIV acquisition mode:** MSM | (reference) |
| **HIV acquisition mode:** IDU | 4.71 (1.59-13.93); p= 0.005 |
| **HIV acquisition mode:** Heterosexual | 0.88 (0.48-1.62); p= 0.675 |
| **HIV acquisition mode:** Other | 2.78 (0.99-7.81); p= 0.052 |
| **Ethnicity:** White | (reference) |
| **Ethnicity:** Black | 7.24 (2.89-18.12); p<0.001 |
| **Ethnicity:** Hispanic | 2.14 (0.55-8.34); p= 0.272 |
| **Ethnicity:** Asian | 9.21 (2.96-28.64); p<0.001 |
| **BMI:** Underweight | 0.67 (0.11-4.24); p= 0.672 |
| **BMI:** Normal | (reference) |
| **BMI:** Overweight | 4.00 (2.38-6.70); p<0.001 |
| **BMI:** Obese | 25.88 (12.80-52.32); p<0.001 |
| **Smoking status**: Never | (reference) |
| **Smoking status**: Current | 0.97 (0.54-1.74); p= 0.907 |
| **Smoking status**: Past | 1.24 (0.73-2.10); p= 0.430 |
| **Hypertension** | 1.28 (0.83-1.97); p= 0.273 |
| **Dyslipidemia** | 2.97 (1.91-4.62); p<0.001 |
| **Hepatitis C seropositivity** | 0.51 (0.21-1.22); p= 0.132 |
| **History of pancreatitis** | 1.07 (0.11-10.64); p= 0.957 |
| **CD4 cell count category**: 0-<200 cells/μL | 1.98 (0.39-10.03); p= 0.411 |
| **CD4 cell count category**: 200-<350 cells/μL | 1.36 (0.70-2.64); p= 0.368 |
| **CD4 cell count category**: 350-<500 cells/μL | 1.21 (0.66-2.23); p= 0.537 |
| **CD4 cell count category**: ≥500 cells/μL | (reference) |
| **Previous AIDS** | 1.42 (0.88-2.28); p= 0.148 |
| **Received third drug ≥1 year in total**: Without ART | 1.84 (0.23-14.78); p= 0.564 |
| **Received third drug ≥1 year in total**: Integrase inhibitor | 2.25 (0.68-7.49); p= 0.186 |
| **Received third drug ≥1 year in total**: Boosted protease inhibitor | (reference) |
| **Received third drug ≥1 year in total**: Efavirenz | 0.69 (0.22-2.14); p= 0.519 |
| **Received third drug ≥1 year in total**: NNRTI other than efavirenz | 0.78 (0.20-3.12); p= 0.727 |
| **Received third drug ≥1 year in total**: Other | 1.92 (0.76-4.83); p= 0.165 |
| **Received stavudine ≥1 year in total** | 1.69 (0.91-3.15); p= 0.095 |
| **Received zidovudine ≥1 year in total** | 2.07 (1.20-3.56); p= 0.009 |
| **Received didanosine ≥1 year in total** | 1.99 (1.05-3.76); p= 0.034 |

**Note.** All data shown apply to the matching date and are number (%) of participants, unless otherwise indicated.

**Abbreviations.** ART, antiretroviral therapy; IDU, intravenous drug use; MSM, men who have sex with men; NNRTI, non-nucleoside reverse transcriptase inhibitor

* latest leukocyte count before matching date

**Supplementary Table 9: Sensitivity Analysis: Diabetes Odds Ratio (95% Confidence Interval) Final Multivariable Model including Family History of Diabetes (available in 388/2764 participants)**

|  | **Multivariable analysis:**  **Diabetes Odds Ratios (OR) and 95% Confidence Intervals** |
| --- | --- |
| 1^st^ (lowest) leukocyte quintile* | (reference) |
| 2nd leukocyte quintile* | 3.22 (0.73-14.20); p= 0.122 |
| 3rd leukocyte quintile* | 5.95 (1.21-29.28); p= 0.028 |
| 4th leukocyte quintile* | 2.34 (0.62-8.88); p= 0.211 |
| 5th (highest) leukocyte quintile* | 4.89 (1.22-19.51); p= 0.025 |
| **HIV acquisition mode:** MSM | (reference) |
| **HIV acquisition mode:** IDU | 4.84 (0.52-45.05); p= 0.166 |
| **HIV acquisition mode:** Heterosexual | 0.73 (0.23-2.37); p= 0.605 |
| **HIV acquisition mode:** Other | 2.80 (0.66-11.97); p= 0.165 |
| **Ethnicity:** White | (reference) |
| **Ethnicity:** Black | 10.79 (2.20-52.96); p= 0.003 |
| **Ethnicity:** Hispanic | 0.94 (0.01-1.60); p= 0.102 |
| **Ethnicity:** Asian | 6.36 (0.83-48.70); p= 0.075 |
| **BMI:** Underweight | 3.28 (0.12-86.50); p= 0.476 |
| **BMI:** Normal | (reference) |
| **BMI:** Overweight | 8.61 (2.60-28.52); p<0.001 |
| **BMI:** Obese | 125.31 (20.53-764.91); p<0.001 |
| **Smoking status**: Never | (reference) |
| **Smoking status**: Current | 1.21 (0.39-3.80); p= 0.742 |
| **Smoking status**: Past | 1.09 (0.41-2.87); p= 0.860 |
| **Hypertension** | 0.81 (0.31-2.10); p= 0.658 |
| **Dyslipidemia** | 6.18 (2.33-16.37); p<0.001 |
| **Hepatitis C seropositivity** | 0.94 (0.22-3.94), p= 0.931 |
| **History of pancreatitis** | 1046234 (0-infinite); p= 0.993 |
| **Family history of DM**: no family history of DM | (reference) |
| **Family history of DM**: family history of DM | 2.91 (1.11-7.67); p= 0.030 |
| **CD4 cell count category**: 0-<200 cells/μL | 24.67 (2.29-265.30); p= 0.0.008 |
| **CD4 cell count category**: 200-<350 cells/μL | 1.37 (0.43-4.40); p= 0.590 |
| **CD4 cell count category**: 350-<500 cells/μL | 2.26 (0.70-7.29); p= 0.172 |
| **CD4 cell count category**: ≥500 cells/μL | (reference) |
| **Previous AIDS** | 0.37 (0.12-1.18); p= 0.094 |
| **Received third drug ≥1 year in total**: Without ART | 0.56 (0.087-3.64); p= 0.546 |
| **Received third drug ≥1 year in total**: Integrase inhibitor | 0.74 (0.12-4.46); p= 0.740 |
| **Received third drug ≥1 year in total**: Boosted protease inhibitor | (reference) |
| **Received third drug ≥1 year in total**: Efavirenz | 0.36 (0.05-2.71); p= 0.321 |
| **Received third drug ≥1 year in total**: NNRTI other than efavirenz | 0.07 (0.01-0.63); p= 0.018 |
| **Received third drug ≥1 year in total**: Other | 0.64 (0.13-3.17); p= 0.582 |
| **Received stavudine ≥1 year in total** | 0.82 (0.09-7.33); p= 0.856 |
| **Received zidovudine ≥1 year in total** | 8.62 (1.47-50.61); p= 0.017 |
| **Received didanosine ≥1 year in total** | 12.37 (1.02-149.74); p= 0.048 |

**Note.** All data shown apply to the matching date and are number (%) of participants, unless otherwise indicated.

**Abbreviations.** ART, antiretroviral therapy; DM, diabetes mellitus; IDU, intravenous drug use; MSM, men who have sex with men; NNRTI, non-nucleoside reverse transcriptase inhibitor

* latest leukocyte count before matching date

**Supplementary Table 10: Sensitivity Analysis: Diabetes Odds Ratio (95% Confidence Interval) Final Multivariable Model including Physical Activity (available in 1857/2764 participants)**

|  | **Multivariable analysis:**  **Diabetes Odds Ratios (OR) and 95% Confidence Intervals** |
| --- | --- |
| 1^st^ (lowest) leukocyte quintile* | (reference) |
| 2nd leukocyte quintile* | 1.87 (1.14-3.06); p= 0.012 |
| 3rd leukocyte quintile* | 1.91 (1.18-3.08); p= 0.008 |
| 4th leukocyte quintile* | 1.62 (1.00-2.64), p= 0.050 |
| 5th (highest) leukocyte quintile* | 3.20 (1.96-5.23), p<0.001 |
| **Physical activity:** less than once a week | (reference) |
| **Physical activity:** more than once a week | 0.67 (0.51-0.89); p= 0.006 |
| **HIV acquisition mode:** MSM | (reference) |
| **HIV acquisition mode:** IDU | 2.23 (1.18-4.21); p= 0.013 |
| **HIV acquisition mode:** Heterosexual | 0.98 (0.67-1.44); p= 0.933 |
| **HIV acquisition mode:** Other | 2.57 (1.36-4.86); p= 0.004 |
| **Ethnicity:** White | (reference) |
| **Ethnicity:** Black | 4.07 (2.25-7.36); p<0.001 |
| **Ethnicity:** Hispanic | 1.47 (0.56-3.82); p= 0.431 |
| **Ethnicity:** Asian | 5.54 (2.72-11.27); p<0.001 |
| **BMI:** Underweight | 1.10 (0.49-2.48); p= 0.810 |
| **BMI:** Normal | (reference) |
| **BMI:** Overweight | 3.02 (2.18-4.16); p<0.001 |
| **BMI:** Obese | 13.83 (8.95-21.38); p<0.001 |
| **Smoking status**: Never | (reference) |
| **Smoking status**: Current | 1.09 (0.75-1.58); p= 0.664 |
| **Smoking status**: Past | 1.24 (0.88-1.74); p= 0.226 |
| **Hypertension** | 1.28 (0.96-1.69); p= 0.090 |
| **Dyslipidemia** | 2.32 (1.77-3.04); p<0.001 |
| **Hepatitis C seropositivity** | 0.68 (0.40-1.15); p= 0.154 |
| **History of pancreatitis** | 5.48 (1.57-19.16); p= 0.008 |
| **CD4 cell count category**: 0-<200 cells/μL | 4.15 (2.08-8.26); p<0.001 |
| **CD4 cell count category**: 200-<350 cells/μL | 1.43 (0.91-2.25); p= 0.119 |
| **CD4 cell count category**: 350-<500 cells/μL | 0.93 (0.63-1.37); p= 0.709 |
| **CD4 cell count category**: ≥500 cells/μL | (reference) |
| **Previous AIDS** | 1.14 (0.84-1.56); p= 0.395 |
| **Received third drug ≥1 year in total**: Without ART | 1.45 (0.59-3.56); p= 0.422 |
| **Received third drug ≥1 year in total**: Integrase inhibitor | 2.19 (1.01-4.75); p= 0.048 |
| **Received third drug ≥1 year in total**: Boosted protease inhibitor | (reference) |
| **Received third drug ≥1 year in total**: Efavirenz | 1.67 (1.01-2.78); p= 0.047 |
| **Received third drug ≥1 year in total**: NNRTI other than efavirenz | 0.69 (0.30-1.55); p= 0.368 |
| **Received third drug ≥1 year in total**: Other | 1.58 (1.03-2.43); |
| **Received stavudine ≥1 year in total** | 1.22 (0.83-1.80); p= 0.304 |
| **Received zidovudine ≥1 year in total** | 1.54 (1.10-2.15); p= 0.013 |
| **Received didanosine ≥1 year in total** | 1.78 (1.20-2.65); p= 0.004 |

**Note.** All data shown apply to the matching date and are number (%) of participants, unless otherwise indicated.

**Abbreviations.** ART, antiretroviral therapy; DM, diabetes mellitus; IDU, intravenous drug use; MSM, men who have sex with men; NNRTI, non-nucleoside reverse transcriptase inhibitor

* latest leukocyte count before matching date

**Supplementary Table 11: Sensitivity Analysis: Diabetes Odds Ratio (95% Confidence Interval) in Multivariable Analysis with Current Exposure (past 6 Months) to Third ART Drug (Instead of (≥1 Year)**

|  | **Multivariable analysis:**  **Diabetes Odds Ratios (OR) and 95% Confidence Intervals** |
| --- | --- |
| 1^st^ (lowest) leukocyte quintile* | (reference) |
| 2nd leukocyte quintile* | 1.46 (1.02-2.08); p= 0.038 |
| 3rd leukocyte quintile* | 1.71 (1.20-2.44); p= 0.003 |
| 4th leukocyte quintile* | 1.51 (1.04-2.18); p= 0.030 |
| 5th (highest) leukocyte quintile* | 2.55 (1.77-3.68); p<0.001 |
| **HIV acquisition mode:** MSM | (reference) |
| **HIV acquisition mode:** IDU | 1.79 (1.10-2.91); p= 0.019 |
| **HIV acquisition mode:** Heterosexual | 1.46 (1.11-1.94); p= 0.008 |
| **HIV acquisition mode:** Other | 2.62 (1.58-4.33); p<0.001 |
| **Ethnicity:** White | (reference) |
| **Ethnicity:** Black | 3.67 (2.32-5.82); p<0.001 |
| **Ethnicity:** Hispanic | 1.56 (0.73-3.30); p=0.248 |
| **Ethnicity:** Asian | 3.92 (2.19-7.03); p<0.001 |
| **BMI:** Underweight | 1.47 (0.85-2.57); p= 0.171 |
| **BMI:** Normal | (reference) |
| **BMI:** Overweight | 2.65 (2.08-3.39); p<0.001 |
| **BMI:** Obese | 10.48 (7.50-14.63); p<0.001 |
| **Smoking status**: Never | (reference) |
| **Smoking status**: Current | 0.95 (0.72-1.26); p= 0.723 |
| **Smoking status**: Past | 1.15 (0.88-1.50); p= 0.316 |
| **Hypertension** | 1.22 (0.97-1.52); p= 0.085 |
| **Dyslipidemia** | 2.24 (1.81-2.76); p<0.001 |
| **Hepatitis C seropositivity** | 0.78 (0.53-1.17); p= 0.230 |
| **History of pancreatitis** | 6.09 (2.19-16.92); p= 0.001 |
| **CD4 cell count category**: 0-<200 cells/μL | 2.48 (1.57-3.91); p<0.001 |
| **CD4 cell count category**: 200-<350 cells/μL | 1.60 (1.16-2.21); p= 0.004 |
| **CD4 cell count category**: 350-<500 cells/μL | 1.15 (0.87-1.54); p= 0.324 |
| **CD4 cell count category**: ≥500 cells/μL | (reference) |
| **Previous AIDS** | 1.09 (0.86-1.39); p= 0.452 |
| **Current third drug (past 6 months)**: Without ART | 1.14 (0.66-1.95); p= 0.644 |
| **Current third drug (past 6 months)**: Integrase inhibitor | 1.88 (1.27-2.79); p= 0.002 |
| **Current third drug (past 6 months)**: Boosted protease inhibitor | (reference) |
| **Current third drug (past 6 months)**: Efavirenz | 1.14 (0.78-1.66); p= 0.498 |
| **Current third drug (past 6 months)**: NNRTI other than efavirenz | 1.11 (0.72-1.70); p= 0.641 |
| **Current third drug (past 6 months)**: Other | 1.57 (1.12-2.19); p= 0.009 |
| **Current exposure (past 6 months)**: Stavudine | 2.02 (1.07-3.82); p= 0.030 |
| **Current exposure (past 6 months)**: Zidovudine | 1.25 (0.87-1.82); p= 0.229 |
| **Current exposure (past 6 months)**: Didanosine | 1.06 (0.62-1.84); p= 0.824 |

**Note.** All data shown apply to the matching date and are number (%) of participants, unless otherwise indicated.

**Abbreviations.** ART, antiretroviral therapy; IDU, intravenous drug use; MSM, men who have sex with men; NNRTI, non-nucleoside reverse transcriptase inhibitor

* latest leukocyte count before matching date

**Supplementary Table 12: Sensitivity Analysis: Diabetes Odds Ratio (95% Confidence Interval) in Multivariable Analysis when Considering Third ART Drug AND Exposure to Any Tenofovir (TDF or TAF) for≥1 Year**

|  | **Multivariable analysis:**  **Diabetes Odds Ratios (OR) and 95% Confidence Intervals** |
| --- | --- |
| 1^st^ (lowest) leukocyte quintile* | (reference) |
| 2nd leukocyte quintile* | 1.42 (0.99-2.03); p= 0.057 |
| 3rd leukocyte quintile* | 1.69 (1.18-2.41); p= 0.004 |
| 4th leukocyte quintile* | 1.47 (1.01-2.14); p= 0.046 |
| 5th (highest) leukocyte quintile* | 2.48 (1.71-3.59); p<0.001 |
| **HIV acquisition mode:** MSM | (reference) |
| **HIV acquisition mode:** IDU | 2.02 (1.23-3.33); p= 0.006 |
| **HIV acquisition mode:** Heterosexual | 1.48 (1.11-1.97); p= 0.007 |
| **HIV acquisition mode:** Other | 2.73 (1.64-4.57); p<0.001 |
| **Ethnicity:** White | (reference) |
| **Ethnicity:** Black | 3.64 (2.29-5.79); p<0.001 |
| **Ethnicity:** Hispanic | 1.46 (0.68-3.15); p= 0.336 |
| **Ethnicity:** Asian | 3.76 (2.07-6.85); p<0.001 |
| **BMI:** Underweight | 1.28 (0.72-2.26); p= 0.401 |
| **BMI:** Normal | (reference) |
| **BMI:** Overweight | 2.82 (2.20-3.62); p<0.001 |
| **BMI:** Obese | 11.56 (8.19-16.31); p<0.001 |
| **Smoking status**: Never | (reference) |
| **Smoking status**: Current | 0.96 (0.72-1.28); p= 0.800 |
| **Smoking status**: Past | 1.16 ( 0.89-1.52); p= 0.277 |
| **Hypertension** | 1.19 (0.95-1.49); p= 0.131 |
| **Dyslipidemia** | 2.24 (1.81-2.77); p<0.001 |
| **Hepatitis C seropositivity** | 0.77 (0.51-1.15 ); p= 0.204 |
| **CD4 cell count category**: 0-<200 cells/μL | 2.64 (1.67-4.18); p<0.001 |
| **CD4 cell count category**: 200-<350 cells/μL | 1.61 (1.16-2.23); p= 0.004 |
| **CD4 cell count category**: 350-<500 cells/μL | 1.15 (0.86-1.54); p= 0.345 |
| **CD4 cell count category**: ≥500 cells/μL | (reference) |
| **Previous AIDS** | 1.11 (0.88-1.41); p= 0.384 |
| **Received third drug ≥1 year in total**: Without ART | 1.59 (0.87-2.89); p= 0.132 |
| **Received third drug ≥1 year in total**: Integrase inhibitor | 2.02 (1.00-4.07); p= 0.051 |
| **Received third drug ≥1 year in total**: Boosted protease inhibitor | (reference) |
| **Received third drug ≥1 year in total**: Efavirenz | 1.66 (1.13-2.43); p= 0.009 |
| **Received third drug ≥1 year in total**: NNRTI other than efavirenz | 0.78 (0.43-1.41); p= 0.404 |
| **Received third drug ≥1 year in total**: Other | 1.63 (1.18-2.25), p= 0.003 |
| **Received stavudine ≥1 year in total** | 1.54 (1.16-2.04); p= 0.003 |
| **Received zidovudine ≥1 year in total** | 1.35 (1.03-1.76); p= 0.029 |
| **Received didanosine ≥1 year in total** | 1.30 (0.98-1.74); p= 0.069 |
| **Received drug ≥1 year in total**: No tenofovir | (reference) |
| **Received drug ≥1 year in total**: TDF | 0.66 (0.50-0.88); p= 0.005 |
| **Received drug ≥1 year in total**: TAF | 1.06 (0.68-1.66); p= 0.793 |

**Note.** All data shown apply to the matching date and are number (%) of participants, unless otherwise indicated.

**Abbreviations.** ART, antiretroviral therapy; IDU, intravenous drug use; MSM, men who have sex with men; NNRTI, non-nucleoside reverse transcriptase inhibitor; TAF, tenofovir alafenamide; TDF, tenofovir disoproxil fumarate

* latest leukocyte count before matching date

**Supplementary Table 13: Sensitivity Analysis: Diabetes Odds Ratio (95% Confidence Interval) in Multivariable Analysis Restricted to Participants without Corticoid Use >3 Months (2260/2764 study participants; 627 cases, 1633 controls)**

|  | **Multivariable analysis:**  **Diabetes Odds Ratios (OR) and 95% Confidence Intervals** |
| --- | --- |
| 1^st^ (lowest) leukocyte quintile* | (reference) |
| 2nd leukocyte quintile* | 1.47 (0.98-2.19); p= 0.062 |
| 3rd leukocyte quintile* | 1.68 (1.13-2.51); p= 0.011 |
| 4th leukocyte quintile* | 1.40 (0.92-2.14); p= 0.119 |
| 5th (highest) leukocyte quintile* | 2.79 (1.83-4.24); p<0.001 |
| **HIV acquisition mode:** MSM | (reference) |
| **HIV acquisition mode:** IDU | 2.33 (1.35-4.04); p= 0.003 |
| **HIV acquisition mode:** Heterosexual | 1.37 (0.99-1.89); p= 0.061 |
| **HIV acquisition mode:** Other | 2.73 (1.52-4.89); p= 0.001 |
| **Ethnicity:** White | (reference) |
| **Ethnicity:** Black | 3.50 (2.09-5.83); p<0.001 |
| **Ethnicity:** Hispanic | 2.03 (0.89-4.63); p= 0.092 |
| **Ethnicity:** Asian | 4.53 (2.38-8.63); p<0.001 |
| **BMI:** Underweight | 1.32 (0.67-2.60); p= 0.427 |
| **BMI:** Normal | (reference) |
| **BMI:** Overweight | 3.02 (2.29-4.00); p<0.001 |
| **BMI:** Obese | 13.62 (9.20-20.17); p<0.001 |
| **Smoking status**: Never | (reference) |
| **Smoking status**: Current | 0.85 (0.62-1.18); p= 0.334 |
| **Smoking status**: Past | 1.08 (0.80-1.45); p= 0.638 |
| **Hypertension** | 1.17 (0.91-1.51); p= 0.208 |
| **Dyslipidemia** | 2.36 (1.85-3.00); p<0.001 |
| **Hepatitis C seropositivity** | 0.79 (0.50-1.25); p= 0.319 |
| **History of pancreatitis** | 4.18 (1.41-12.46); p= 0.010 |
| **CD4 cell count category**: 0-<200 cells/μL | 2.53 (1.41-4.54); p= 0.002 |
| **CD4 cell count category**: 200-<350 cells/μL | 1.52 (1.05-2.20); p= 0.028 |
| **CD4 cell count category**: 350-<500 cells/μL | 1.21 (0.88-1.66); p= 0.246 |
| **CD4 cell count category**: ≥500 cells/μL | (reference) |
| **Previous AIDS** | 1.10 (0.84-1.44); p= 0.493 |
| **Received third drug ≥1 year in total**: Without ART | 2.20 (1.10-4.42); p= 0.026 |
| **Received third drug ≥1 year in total**: Integrase inhibitor | 2.53 (1.19-5.36); p= 0.016 |
| **Received third drug ≥1 year in total**: Boosted protease inhibitor | (reference) |
| **Received third drug ≥1 year in total**: Efavirenz | 1.84 (1.19-2.83); p= 0.006 |
| **Received third drug ≥1 year in total**: NNRTI other than efavirenz | 1.02 (0.54-1.95); p= 0.942 |
| **Received third drug ≥1 year in total**: Other | 2.05 (1.42-2.96); p<0.001 |
| **Received stavudine ≥1 year in total** | 1.38 (1.01-1.89); p= 0.045 |
| **Received zidovudine ≥1 year in total** | 1.46 (1.09-1.97); p= 0.012 |
| **Received didanosine ≥1 year in total** | 1.30 (0.95-1.79); p= 0.101 |

**Note.** All data shown apply to the matching date and are number (%) of participants, unless otherwise indicated.

**Abbreviations.** ART, antiretroviral therapy; IDU, intravenous drug use; MSM, men who have sex with men; NNRTI, non-nucleoside reverse transcriptase inhibitor

* latest leukocyte count before matching date

**Supp Table 14: Sensitivity Analysis: Diabetes Odds Ratio (95% Confidence Interval) in Multivariable Analysis considering “Statin exposure” rather than “Dyslipidemia”**

|  | **Multivariable analysis:**  **Diabetes Odds Ratios (OR) and 95% Confidence Intervals** |
| --- | --- |
| 1^st^ (lowest) leukocyte quintile* | (reference) |
| 2nd leukocyte quintile* | 1.43 (1.00-2.03); p= 0.048 |
| 3rd leukocyte quintile* | 1.71 (1.20-2.42); p= 0.003 |
| 4th leukocyte quintile* | 1.49 (1.03-2.15); p= 0.034 |
| 5th (highest) leukocyte quintile* | 2.47 (1.71-3.56); p<0.001 |
| **HIV acquisition mode:** MSM | (reference) |
| **HIV acquisition mode:** IDU | 1.91 (1.18-3.10); p= 0.008 |
| **HIV acquisition mode:** Heterosexual | 1.47 (1.11-1.95); p= 0.007 |
| **HIV acquisition mode:** Other | 3.06 (1.84-5.08); p<0.001 |
| **Ethnicity:** White | (reference) |
| **Ethnicity:** Black | 3.48 (2.20-5.49); p<0.001 |
| **Ethnicity:** Hispanic | 1.41 (0.67-3.00); p= 0.368 |
| **Ethnicity:** Asian | 3.78 (2.10-6.79); p<0.001 |
| **BMI:** Underweight | 1.17 (0.67-2.05); p= 0.582 |
| **BMI:** Normal | (reference) |
| **BMI:** Overweight | 3.01 (2.36-3.84); p<0.001 |
| **BMI:** Obese | 12.07 (8.64-16.86); p<0.001 |
| **Smoking status**: Never | (reference) |
| **Smoking status**: Current | 0.94 (0.71-1.24); p= 0.645 |
| **Smoking status**: Past | 1.11 (0.85-1.45); p= 0.424 |
| **Hypertension** | 1.21 (0.97-1.51); p= 0.092 |
| **Statin exposure** | 1.71 (1.14-2.57); p= 0.010 |
| **Hepatitis C seropositivity** | 0.80 (0.54-1.18); p= 0.255 |
| **History of pancreatitis** | 6.43 (2.36-17.54); p<0.001 |
| **CD4 cell count category**: 0-<200 cells/μL | 2.38 (1.52-3.73); p<0.001 |
| **CD4 cell count category**: 200-<350 cells/μL | 1.58 (1.15-2.17); p= 0.005 |
| **CD4 cell count category**: 350-<500 cells/μL | 1.11 (0.83-1.47); p= 0.478 |
| **CD4 cell count category**: ≥500 cells/μL | (reference) |
| **Previous AIDS** | 1.16 (0.92-1.46); p= 0.222 |
| **Received third drug ≥1 year in total**: Without ART | 1.86 (1.05-3.30); p= 0.034 |
| **Received third drug ≥1 year in total**: Integrase inhibitor | 2.21 (1.10-4.45); p= 0.027 |
| **Received third drug ≥1 year in total**: Boosted protease inhibitor | (reference) |
| **Received third drug ≥1 year in total**: Efavirenz | 1.60 (1.11-2.33); p= 0.012 |
| **Received third drug ≥1 year in total**: NNRTI other than efavirenz | 0.81 (0.45-1.44); p= 0.465 |
| **Received third drug ≥1 year in total**: Other | 1.66 (1.21-2.27); p= 0.002 |
| **Received stavudine ≥1 year in total** | 1.59 (1.20-2.09); p= 0.001 |
| **Received zidovudine ≥1 year in total** | 1.36 (1.05-1.77); p= 0.019 |
| **Received didanosine ≥1 year in total** | 1.28 (0.97-1.68); p= 0.086 |

**Note.** All data shown apply to the matching date and are number (%) of participants, unless otherwise indicated.

**Abbreviations.** ART, antiretroviral therapy; IDU, intravenous drug use; MSM, men who have sex with men; NNRTI, non-nucleoside reverse transcriptase inhibitor

* latest leukocyte count before matching date

**Supplementary Figure 1: Proportion of Controls and Cases in the Five Leukocyte Quintiles, and Distribution of Leukocyte Count in 2032 Diabetes-Free Controls (white bars) and in 732 Cases with Diabetes (gray bars).**


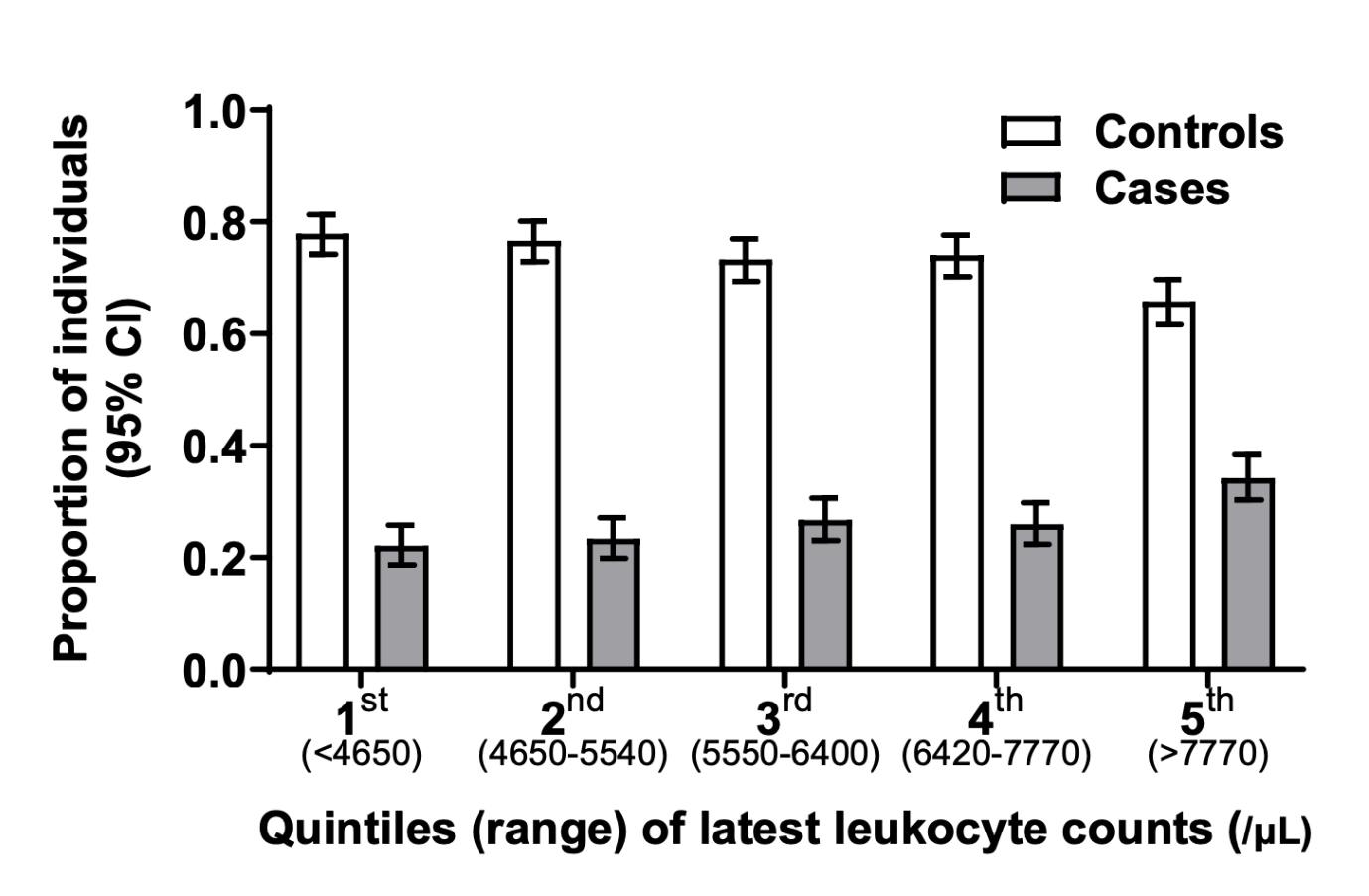


Cases with DM and DM-free controls were divided into 5 quintiles according to their latest leukocyte count prior to the matching date. Here we show number, percentage and 95% confidence intervals of participants in each quintile, plus the range of leukocyte counts in each quintile. There were 122 (22.1%) cases vs. 430 (77.9%) controls in the first quintile, 129 (23.4%) vs. 423 (76.6%) in the second quintile, 148 (26.7%) vs. 406 (73.3%) in the third quintile, 143 (26.0%) vs. 408 (74.0%) in the fourth quintile, and 190 (34.2%) vs. 365 (65.8%) in the fifth quintile. **Supplementary Table 2** shows leukocytes in each quintile in cases and controls separately.

**Abbreviations**: CI, confidence interval.

**Supplementary Figure 2A-B: Descriptive Longitudinal Trends for Leukocyte Count and HIV RNA in Cases and Controls.**


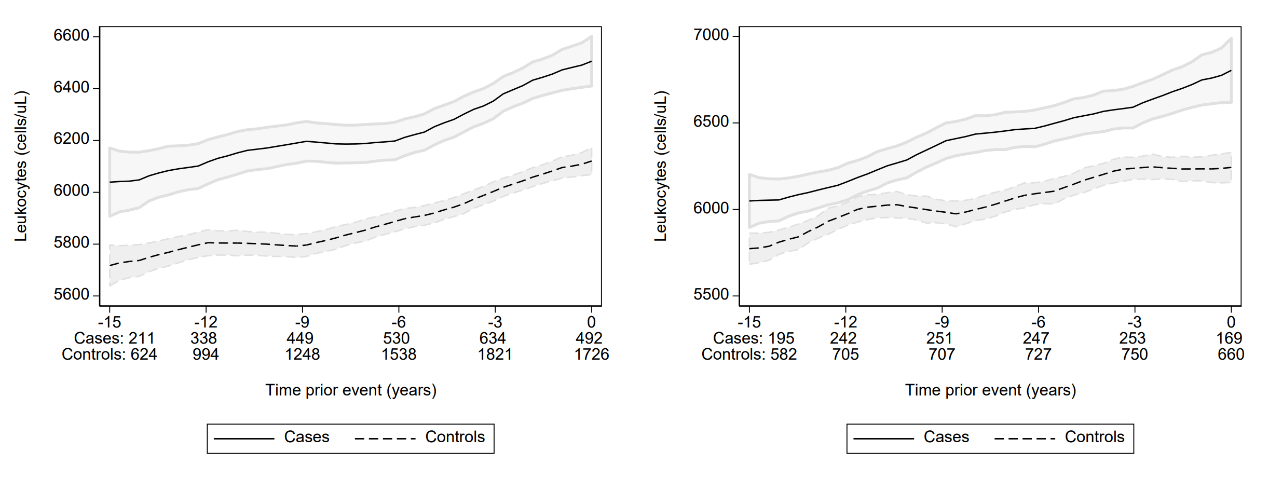
**A: Total Leukocyte Count**


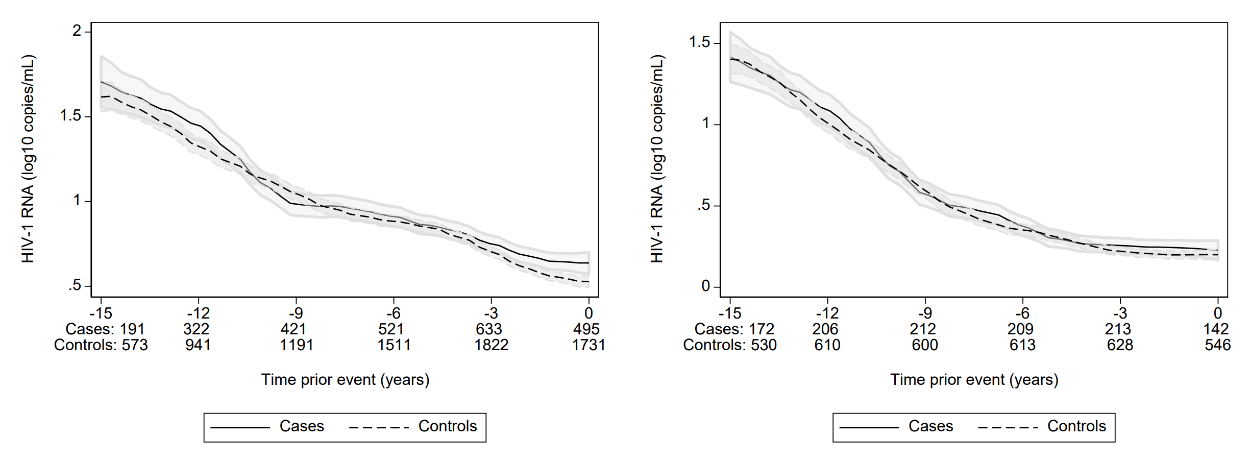
**B: HIV RNA**

Descriptive (observed) trajectories over time for controls vs. cases. Panel A shows leukocytes, and panel B shows HIV RNA. Depicted are counts (line) and 95% confidence intervals (shaded areas), which were created with local polynomial smoothing. Only parameters from regular (per protocol) 6-monthly follow-up SHCS visits up until the matching date were considered. Data of all participants irrespective of observation duration are shown in the graphs on the lefthand side (open cohort design) and data of participants with >15 years observation time only are shown in the graphs on the righthand side (closed cohort design).

**Abbreviations**: SHCS, Swiss HIV Cohort Study
